# ﻿Three new species of the genus *Biasticus* Stål, 1867 (Insecta, Heteroptera, Reduviidae, Harpactorinae) from Central Highlands, Vietnam

**DOI:** 10.3897/zookeys.1118.83156

**Published:** 2022-08-24

**Authors:** Ngoc Linh Ha, Xuan Lam Truong, Tadashi Ishikawa, Weeyawat Jaitrong, Chi Feng Lee, Bounsanong Chouangthavy, Katsuyuki Eguchi

**Affiliations:** 1 Department of Biological Sciences, Graduate School of Science, Tokyo Metropolitan University, Minami-osawa 1-1, Hachioji, Tokyo 192-0397, Japan; 2 Institute of Ecology and Biological Resources, Vietnam Academy of Science and Technology, 18 Hoang Quoc Viet Road, Nghia Do, Cau Giay, Hanoi, Vietnam; 3 Graduate University of Science and Technology, Vietnam Academy of Science and Technology, 18 Hoang Quoc Viet Road, Nghia Do, Cau Giay, Hanoi, Vietnam; 4 Faculty of Agriculture, Tokyo University of Agriculture, Atsugi, Kanagawa, Japan; 5 Office of Natural Science Research, National Science Museum, 39 Moo 3, Khlong 5, Khlong Luang, Pathum Thani, 12120 Thailand; 6 Applied Zoology Division, Taiwan Agricultural Research Institute, Taichung 413, Taiwan; 7 Plant Protection Unit, Department of Plant Science, Faculty of Agriculture, National University of Laos, P.O. Box 7322, Vientiane, Lao People's Democratic Republic

**Keywords:** Assassin bugs, Indo-China, molecular phylogeny, morphometry, taxonomy

## Abstract

Three new assassin bug species of the genus *Biasticus* Stål, 1867 are recognized in Vietnam based on morphological examination, morphometric and molecular phylogenetic analyses, and described as *Biasticustaynguyenensis* Ha, Truong & Ishikawa, **sp. nov.**, *Biasticusgriseocapillus* Ha, Truong & Ishikawa, **sp. nov.**, and *Biasticusluteicollis* Ha, Truong & Ishikawa, **sp. nov.** The conspecific male and female associations of the new species were confirmed by phylogenetic analyses and DNA barcoding of the mitochondrial 16S rDNA and COI genes. All three new species are presently restricted to the Central Highlands, Vietnam (Kon Chu Rang NR, Gia Lai Province, and Chu Yang Sin NP, Dak Lak Province).

## ﻿Introduction

The assassin bug genus *Biasticus* Stål, 1867 was established by monotypy with *Reduviusimpiger* Stål, 1863, which was described based on a single female specimen from Cambodia and is currently assigned to the subfamily Harpactorinae of the family Reduviidae (Stål 1863, 1867; Maldonado 1990). *Biasticus* currently includes 20 valid named species known exclusively from the Oriental and Sino-Japanese realms (Stål 1863; Reuter 1887; Distant 1903; Bergroth 1913; Matsumura 1913; Miller 1941, 1948, 1949, 1954a, 1954b; Hsiao 1979; Hsiao and Ren 1981; Cai and Yang 2002; Ishikawa 2003; Afzal and Ahmad 2019). There has been no notable modification to the genus classification since Hsiao and Ren (1981) added three new species. According to Truong et al. (2015), three species have been recorded from Vietnam, i.e., *B.confusus* Hsiao et al., 1979, *B.flavinotus* (Matsumura, 1913), and *B.flavus* (Distant, 1903).

We discovered dozens of *Biasticus* specimens in Central Highlands, Vietnam, during recent field surveys and examinations of Reduviidae specimens owned by research organizations in Vietnam, Thailand, Laos, Taiwan, and Japan, but they were not identifiable as valid species of the genus. Therefore, this study aims to confirm the taxonomic status of those *Biasticus* specimens using an integrated approach that includes morphological examination, morphometric analyses, molecular phylogenetic analyses, and molecular-based species delimitation analyses, i.e., Assemble Species by Automatic Partitioning (ASAP) (Puillandre et al. 2021) and Bayesian implementation of the Poisson Tree Processes model (bPTP) (Zhang et al. 2013) for species delimitation, as well as the necessary taxonomic treatments.

## ﻿Materials and methods

### ﻿Material examined

This study included 31 specimens (10 male and 21 female adults) collected from Central Highlands, Vietnam (Kon Chu Rang Nature Reserve, Gia Lai Province, and Chu Yang Sin National Park, Dak Lak Province), which are in accordance with the diagnosis of *Biasticus* but were not assigned to any validly named species. The following species that were previously recorded from Vietnam were also herein examined: *B.confusus* Hsiao et al., 1979 (six specimens from Northern Vietnam), *B.flavinotus* (Matsumura, 1913) (eight from Northern Thailand, Northern Vietnam, and Taiwan, including the lectotype), and *B.flavus* (Distant, 1903) (11 from Northern Laos and Northern Thailand). Furthermore, specimens of *Sphedanolestespubinotus* Reuter, 1881, *Rhynocorismendicus* (Stål, 1867), and *Coranus* sp. collected from Vietnam were used as outgroups in molecular phylogenetic analysis (Table 1). The voucher samples of this investigation are housed in the following institutions:

**HU** Matsumura Collection at the Laboratory of Systematic Entomology, Department of Agriculture, Hokkaido University, Sapporo, Japan;

**IEBR** Institute of Ecology and Biological Resources, Vietnam Academy of Science and Technology, Vietnam;

**NSMT** National Museum of Nature and Science, Tokyo, Japan;

**NSM** Department of Entomology, Zoological Research Division, Office of Natural Science Research, National Science Museum, Thailand;

**NUOL-FA** Faculty of Agriculture, National University of Laos, Laos P.D.R.;

**TARI-AZ** Applied Zoology Division, Taiwan Agricultural Research Institute, Taiwan;

**VNMN** Vietnam National Museum of Nature, Vietnam Academy of Science and Technology, Vietnam.

This study followed the description of the genus *Biasticus* slightly modified by Distant (1904): “Body elongate; head subelongate, almost as long as the pronotum, postocular a little longer than anteocular area; rostrum with the first segment shorter than second, a little longer than anteocular area of head; first segment of antennae a little longer than pronotum; anterior lobe of pronotum longitudinally impressed, posterior lobe with a distinct, central, anterior, longitudinally elevation; scutellum not apically produced; hemelytra passing the abdominal apex; legs moderately long and slender; femora apically moderately nodulose, anterior femora very slightly incrassated.”

Examination at the species level was executed by referring to the original descriptions and other taxonomic publications (Stål 1863; Reuter 1887; Distant 1903; Bergroth 1913; Matsumura 1913; Miller 1941, 1948, 1949, 1954a, 1954b; Hsiao 1979; Hsiao and Ren 1981; Cai and Yang 2002; Ishikawa 2003; Afzal and Ahmad 2019) of the following congeners known from Vietnam and adjacent areas: *B.abdominalis* (Reuter, 1887), type location: India and Myanmar; *B.abjectus* Miller, 1941, Borneo; *B.breddin* Miller, 1948, Indonesia; *B.chersonesus* (Distant, 1903), Malaysia and Myanmar; *B.confusus* Hsiao et al., 1979, South China (see also Table 1); *B.dilectus* Miller, 1954, Indonesia; *B.eburneus* Miller, 1941, Borneo; *B.flavinotus* (Matsumura, 1913), Taiwan (see also Table 1); *B.flavus* (Distant, 1903), Hong Kong and Myanmar (see also Table 1); *B.fuliginosus* Reuter, 1887, North India; *B.gagatinus* Breddin, 1903, Indonesia; *B.horfieldi* Distant, 1903, Indonesia; *B.impiger* (Stål, 1863), Cambodia; *B.insignis* (Miller, 1941), Indonesia; *B.lutescens* Breddin, 1903, Indonesia; *B.moultoni* Bergroth, 1913, Malaysia; *B.nigricollis* (Dallas, 1850), Indonesia; *B.obfuscatus* Miller, 1949, Malaysia; *B.princeps* Miller, 1949, Malaysia; *B.ventralis* Hsiao et al., 1979, South China.

Newly obtained specimens were labeled with their specimen IDs and locality information before being individually preserved in vials containing 99% ethanol. Each specimen’s right hind leg was cut off in the lab and used for DNA extraction (then for molecular phylogenetic analysis and DNA barcoding). The rest of the body was pinned or preserved in 99% ethanol for morphological and morphometric study.

**Table 1.** The data of specimens used in this study. Abbreviations and symbols: n/a: no data; HU, Matsumura Collection at the Laboratory of Systematic Entomology, Department of Agriculture, Hokkaido University, Sapporo, Japan; IEBR, Institute of Ecology and Biological Resources, Vietnam Academy of Science and Technology, Vietnam; NSMT, National Museum of Nature and Science, Tokyo, Japan; NSM, Department of Entomology, Zoological Research Division, Office of Natural Science Research, National Science Museum, Thailand; NUOL-FA, Faculty of Agriculture, National University of Laos, Laos P.D.R; TARI-AZ, Applied Zoology Division, Taiwan Agricultural Research Institute Insect Collection, Taiwan Agricultural Research Institute, Taiwan; VNMN, Vietnam National Museum of Nature, Vietnam Academy of Science and Technology, Vietnam; *, tentatively held by HNL (first author); bA–bF, morphospecies code (see in the text).

Morphospecies	Specimen code	Collecting date	Locality	Sex	Accession numbers			Depository
					16S	Uni-Minibar (COI)	COI	
***Biasticus* (ingroups)**								
*B.taynguyenensis* Ha, Truong & Ishikawa, sp. nov. [bA]	HNL2018-036	09, v, 2018	Vietnam, Dak Lak	♀	OM908207	ON542864	OM868188	IEBR*
*B.taynguyenensis* Ha, Truong & Ishikawa, sp. nov. [bA]	HNL2018-072	08, v, 2018	Vietnam, Gia Lai	♀	OM908210	ON542867	OM868178	IEBR*
*B.taynguyenensis* Ha, Truong & Ishikawa, sp. nov. [bA]	HNL2018-073	08, v, 2018	Vietnam, Gia Lai	♀	OM908211	ON542868	OM868192	NSMT
*B.taynguyenensis* Ha, Truong & Ishikawa, sp. nov. [bA]	HNL2018-074	08, v, 2018	Vietnam, Gia Lai	♀	OM908212	ON542869	OM868193	NSMT
*B.taynguyenensis* Ha, Truong & Ishikawa, sp. nov. [bA]	HNL2018-075	08, v, 2018	Vietnam, Gia Lai	♀	OM908213	ON542870	OM868194	VNMN
*B.taynguyenensis* Ha, Truong & Ishikawa, sp. nov. [bA]	HNL2018-076	08, v, 2018	Vietnam, Gia Lai	♀	ON554765	ON542871	n/a	IEBR
*B.taynguyenensis* Ha, Truong & Ishikawa, sp. nov. [bA]	TXL2016-545	28, iv, 2016	Vietnam, Gia Lai	♂	OM908227	ON542894	OM868177	NSMT
*B.griseocapillus* Ha, Truong & Ishikawa, sp. nov. [bB]	HNL2018-007	05, v, 2018	Vietnam, Gia Lai	♀	OM908197	ON542854	OM868176	IEBR*
*B.griseocapillus* Ha, Truong & Ishikawa, sp. nov. [bB]	HNL2018-037	09, v, 2018	Vietnam, Dak Lak	♀	OM908208	ON542865	OM868189	VNMN
*B.griseocapillus* Ha, Truong & Ishikawa, sp. nov. [bB]	HNL2018-038	09, v, 2018	Vietnam, Dak Lak	♀	OM908209	ON542866	OM868191	NSMT
*B.griseocapillus* Ha, Truong & Ishikawa, sp. nov. [bB]	TXL2016-546	28, iv, 2016	Vietnam, Gia Lai	♂	OM908228	ON542895	OM868190	NSMT
*B.luteicollis* Ha, Truong & Ishikawa, sp. nov. [bC]	HNL2018-017	09, v, 2018	Vietnam, Dak Lak	♀	OM908198	ON542855	OM868179	VNMN
*B.luteicollis* Ha, Truong & Ishikawa, sp. nov. [bC]	HNL2018-018	09, v, 2018	Vietnam, Dak Lak	♀	OM908199	ON542856	OM868180	IEBR*
*B.luteicollis* Ha, Truong & Ishikawa, sp. nov. [bC]	HNL2018-019	09, v, 2018	Vietnam, Dak Lak	♀	OM908200	ON542857	OM868181	IEBR*
*B.luteicollis* Ha, Truong & Ishikawa, sp. nov. [bC]	HNL2018-020	09, v, 2018	Vietnam, Dak Lak	♀	OM908201	ON542858	OM868182	IEBR*
*B.luteicollis* Ha, Truong & Ishikawa, sp. nov. [bC]	HNL2018-021	09, v, 2018	Vietnam, Dak Lak	♀	OM908202	ON542859	OM868183	NSMT
*B.luteicollis* Ha, Truong & Ishikawa, sp. nov. [bC]	HNL2018-022	09, v, 2018	Vietnam, Dak Lak	♂	OM908203	ON542860	OM868184	NSMT
*B.luteicollis* Ha, Truong & Ishikawa, sp. nov. [bC]	HNL2018-023	09, v, 2018	Vietnam, Dak Lak	♀	OM908204	ON542861	OM868185	NSMT
*B.luteicollis* Ha, Truong & Ishikawa, sp. nov. [bC]	HNL2018-024	09, v, 2018	Vietnam, Dak Lak	♀	OM908205	ON542862	OM868186	NSMT
*B.luteicollis* Ha, Truong & Ishikawa, sp. nov. [bC]	HNL2018-025	09, v, 2018	Vietnam, Dak Lak	♂	OM908206	ON542863	OM868187	NSMT
*B.luteicollis* Ha, Truong & Ishikawa, sp. nov. [bC]	HNL2018-078	08, v, 2018	Vietnam, Gia Lai	♀	OM908214	ON542872	OM868195	IEBR*
*B.luteicollis* Ha, Truong & Ishikawa, sp. nov. [bC]	HNL2018-079	08, v, 2018	Vietnam, Gia Lai	♂	OM908215	ON542873	OM868196	IEBR*
*B.luteicollis* Ha, Truong & Ishikawa, sp. nov. [bC]	HNL2018-080	08, v, 2018	Vietnam, Gia Lai	♀	OM908216	ON542874	OM868197	IEBR*
*B.luteicollis* Ha, Truong & Ishikawa, sp. nov. [bC]	HNL2018-081	08, v, 2018	Vietnam, Gia Lai	♀	OM908217	ON542875	OM868198	IEBR*
*B.luteicollis* Ha, Truong & Ishikawa, sp. nov. [bC]	HNL2018-082	08, v, 2018	Vietnam, Gia Lai	♀	OM908218	ON542876	OM868199	IEBR*
*B.luteicollis* Ha, Truong & Ishikawa, sp. nov. [bC]	HNL2018-083	08, v, 2018	Vietnam, Gia Lai	♂	OM908219	ON542877	OM868200	IEBR*
*B.luteicollis* Ha, Truong & Ishikawa, sp. nov. [bC]	HNL2018-084	08, v, 2018	Vietnam, Gia Lai	♂	OM908220	ON542878	OM868201	IEBR*
*B.luteicollis* Ha, Truong & Ishikawa, sp. nov. [bC]	HNL2018-085	08, v, 2018	Vietnam, Gia Lai	♂	OM908221	ON542879	OM868202	IEBR*
*B.luteicollis* Ha, Truong & Ishikawa, sp. nov. [bC]	HNL2018-086	08, v, 2018	Vietnam, Gia Lai	♂	OM908222	ON542880	OM868203	NSMT
*B.luteicollis* Ha, Truong & Ishikawa, sp. nov. [bC]	TXL2016-616	05, v, 2018	Vietnam, Dak Lak	♀	OM908229	ON542896	OM868208	IEBR
*B.luteicollis* Ha, Truong & Ishikawa, sp. nov. [bC]	TXL2016-617	05, v, 2018	Vietnam, Dak Lak	♂	OM908230	ON542897	OM868209	IEBR
*B.confusus* Hsiao et al., 1979 [bE]	NSMT-I-He-8263	15.v.1998	Vietnam, Cao Bang	♂	n/a	n/a	n/a	NSMT
*B.confusus* Hsiao et al., 1979 [bE]	NSMT-I-He-73786	18.v.2003	Vietnam, Lao Cai	♂	n/a	n/a	n/a	NSMT
*B.confusus* Hsiao et al., 1979 [bE]	VN-Hem-1998-010	15.v.1998	Vietnam, Cao Bang	♀	ON554779	ON542898	n/a	IEBR*
*B.confusus* Hsiao et al., 1979 [bE]	VN-Hem-1998-011	15.v.1998	Vietnam, Cao Bang	♀	n/a	ON542899	n/a	IEBR*
*B.confusus* Hsiao et al., 1979 [bE]	VN-Hem-1998-012	22-27.v.1998	Vietnam, Cao Bang	♀	n/a	ON542900	n/a	IEBR*
*B.confusus* Hsiao et al., 1979 [bE]	ADNg2020-027	6.vi.2020	Vietnam, Cao Bang		ON554766	ON542882	n/a	IEBR*
*B.flavinotus* (Matsumura, 1913) [bD] (lectotype)			Taiwan, Gyochi (Yuechih)	♀	n/a	n/a	n/a	HU
*B.flavinotus* (Matsumura, 1913) [bD]		iv-v.1928	Taiwan	♂	n/a	n/a	n/a	HU
*B.flavinotus* (Matsumura, 1913) [bD]	HNL2018-117	12, v, 2018	Vietnam, Lang Son	♀	OM908223	ON542881	OM868204	IEBR*
*B.flavinotus* (Matsumura, 1913) [bD]	TXL2000-006	20, x, 2000	Vietnam, Son La	♀	n/a	n/a	n/a	IEBR
*B.flavinotus* (Matsumura, 1913) [bD]	HEM-TH1999-002	25, iii, 1999	Thailand, Chiang Mai	♀	n/a	n/a	n/a	NSM
*B.flavinotus* (Matsumura, 1913) [bD]	HEM-TH2004-022	3, vi, 2004	Thailand, Chiang Rai	♀	n/a	ON542892	n/a	NSM
*B.flavinotus* (Matsumura, 1913) [bD]	TW-Redu-2014-001	3, vii, 2014	Taiwan, Nantou	♀	ON554776	ON542893	n/a	TARI-AZ
*B.flavinotus* (Matsumura, 1913) [bD]	TW-Redu-2019-001	3, v, 2019	Taiwan, Taitung	♀	ON554777	n/a	n/a	TARI-AZ
*B.flavus* (Distant, 1903) [bF]	LA-Redu-2004-006	15, v, 2004	Laos, Houaphan	♀	ON554778	ON542883	n/a	NUOL-FA
*B.flavus* (Distant, 1903) [bF]	LA-Redu-2004-011	21, v, 2004	Laos, Houaphan	♂	n/a	n/a	n/a	NUOL-FA
*B.flavus* (Distant, 1903) [bF]	LA-Redu-2004-014	22, v, 2004	Laos, Houaphan	♂	n/a	n/a	n/a	NUOL-FA
*B.flavus* (Distant, 1903) [bF]	HEM-TH2004-016	24, v, 2004	Thailand, Chiang Mai	♂	ON554770	ON542887	n/a	NSM
*B.flavus* (Distant, 1903) [bF]	HEM-TH2004-017	24, v, 2004	Thailand, Chiang Mai	♀	ON554771	ON542888	n/a	NSM
*B.flavus* (Distant, 1903) [bF]	HEM-TH2004-018	25, v, 2004	Thailand, Chiang Mai	♂	ON554772	ON542889	n/a	NSM
*B.flavus* (Distant, 1903) [bF]	HEM-TH2004-019	25, v, 2004	Thailand, Chiang Mai	♂	ON554773	ON542890	n/a	NSM
*B.flavus* (Distant, 1903) [bF]	HEM-TH2004-021	3, vi, 2004	Thailand, Chiang Rai	♀	ON554774	ON542891	n/a	NSM
*B.flavus* (Distant, 1903) [bF]	LA-Redu-2008-005	4, v, 2008	Laos, Xieng Khouang	♀	ON554767	ON542884	n/a	NUOL-FA
*B.flavus* (Distant, 1903) [bF]	LA-Redu-2010-004	11, v, 2010	Laos, Xieng Khouang	♀	ON554768	ON542885	n/a	NUOL-FA
*B.flavus* (Distant, 1903) [bF]	LA-Redu-2010-005	11, v, 2010	Laos, Xieng Khouang	♂	ON554769	ON542886	n/a	NUOL-FA
**Outgroups**								
*Sphedanolestespubinotus* Reuter, 1881	HNL2019-002	11, iii, 2019	Vietnam, Quang Tri	♀	OP106594	OP103647	n/a	IEBR
*Rhynocorismendicus* (Stål, 1867)	HNL2018-040	9, v, 2018	Vietnam, Gia Lai	♂	OP106592	OP103646	n/a	IEBR*
*Coranus* sp.	TXL2018-128	15, vi, 2018	Vietnam, Son La	♀	OP106593	OP103648	n/a	IEBR*

### ﻿Morphological examination and imaging

External structural characteristics were observed for dry-mounted and ethanol-preserved specimens using a Nikon SMZ1270 stereomicroscope. The genitalia were prepared for examination as described below. Firstly, each male specimen was relaxed by soaking for 3 days in 70% ethanol. After that, the male genitalia was detached from the body and then soaked in hot 10% KOH for five minutes until body fat and muscle was released. The endosoma was pulled out of the phallosoma by fine tweezers after removing the phallus from the pygophore. All parts of male genitalia were preserved in a genitalia vial filled with propylene glycol and subsequently associated with the pinned specimens. Next, the female genitalia were inspected without being detached from the body. A Nikon SMZ1270 stereomicroscope was used to examine the male and female genital morphology.

Focus stacking was executed using Helicon Focus Pro 7.5.3 software (Helicon Soft Ltd., Ukraine) based on a sequence of the source pictures photographed by a Canon EOS Kiss X9 digital camera connected to a Nikon AZ100 stereomicroscope, and artifacts were removed using the retouch function of the software. After that, the contrast, brightness, color balance, and intensity were adjusted using Adobe Photoshop Elements 10.0 software (Adobe Systems Incorporated, San Jose, CA, USA) using a color corresponding sticker (CASMATCH, Bear Medic Corporation, Japan).

### ﻿Measurement, indices, and terminology

**Figure 1.**
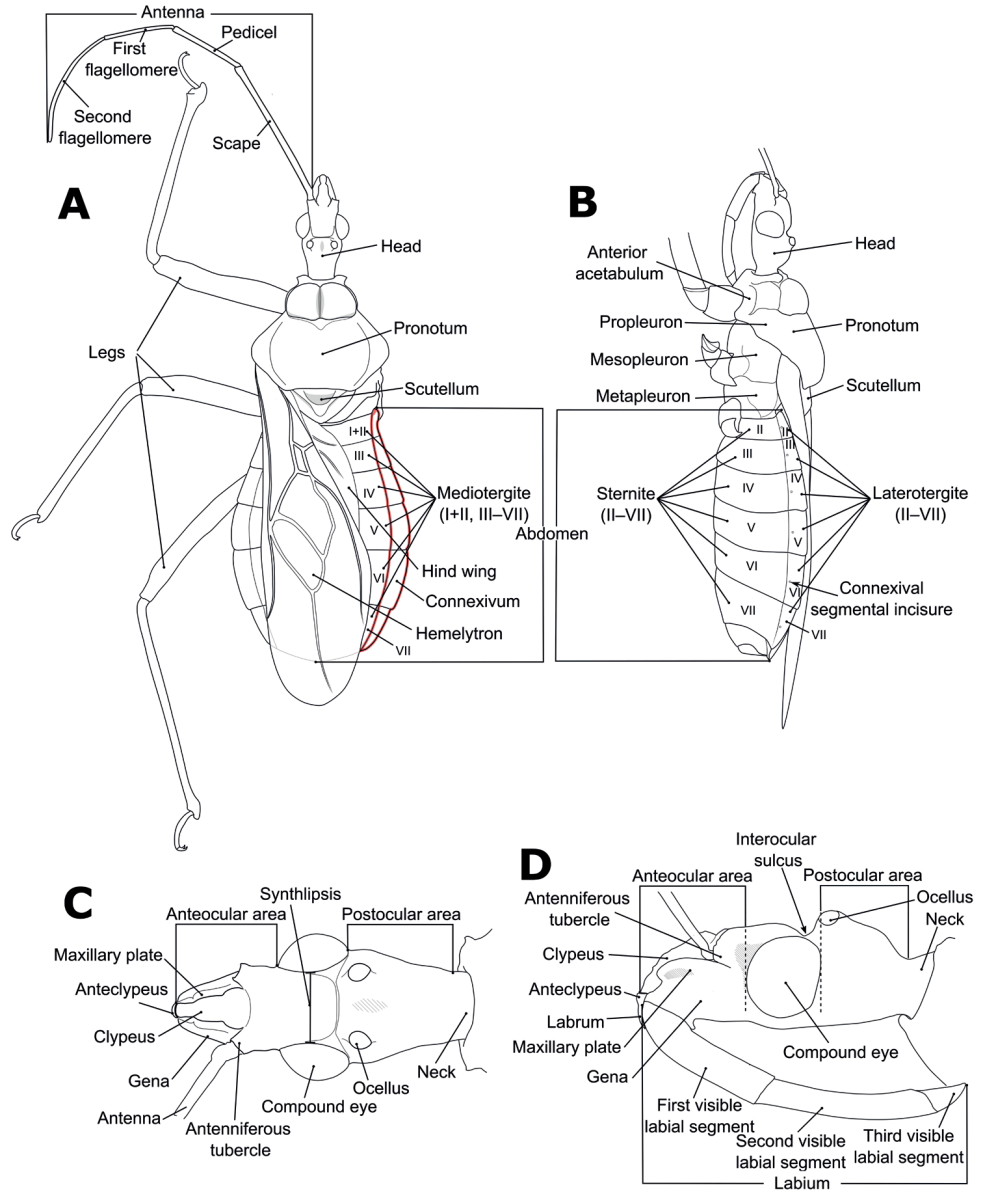
Structure and morphological terms of *Biasticus* species. Drawing based on *Biasticusluteicollis* Ha, Truong & Ishikawa, sp. nov., paratype, ♀, HNL2018-024 **A** 4 body in dorsal view **B** body in lateral view **C** head in dorsal view **D** head in lateral view.

**Figure 2.**
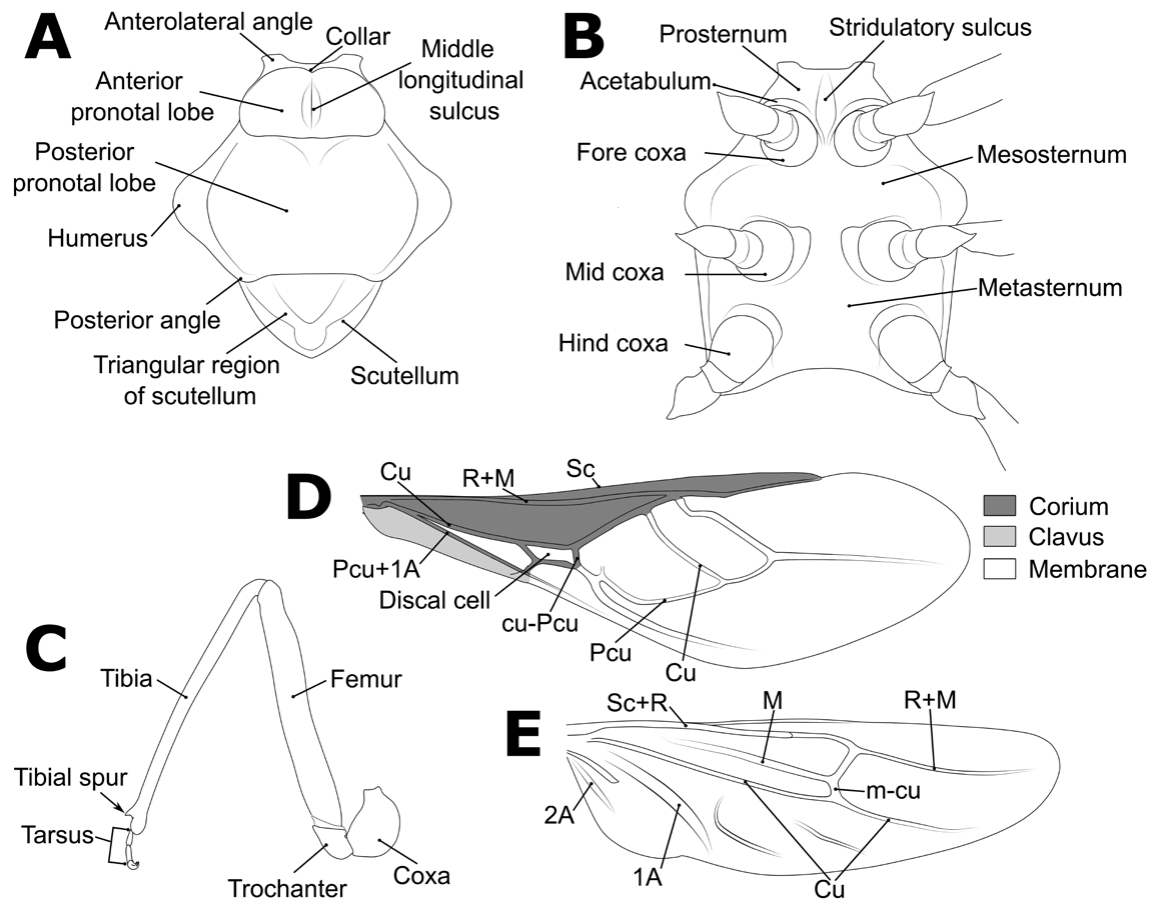
Structure and morphological terms of *Biasticus* species. Drawing based on *Biasticusluteicollis* Ha, Truong & Ishikawa, sp. nov., paratype, ♀, HNL2018-024 **A** pronotum and scutellum in dorsal view **B** thorax in ventral view **C** right fore leg in anterior view **D** right hemelytron in dorsal view **E** right hind wing in dorsal view.

**Figure 3.**
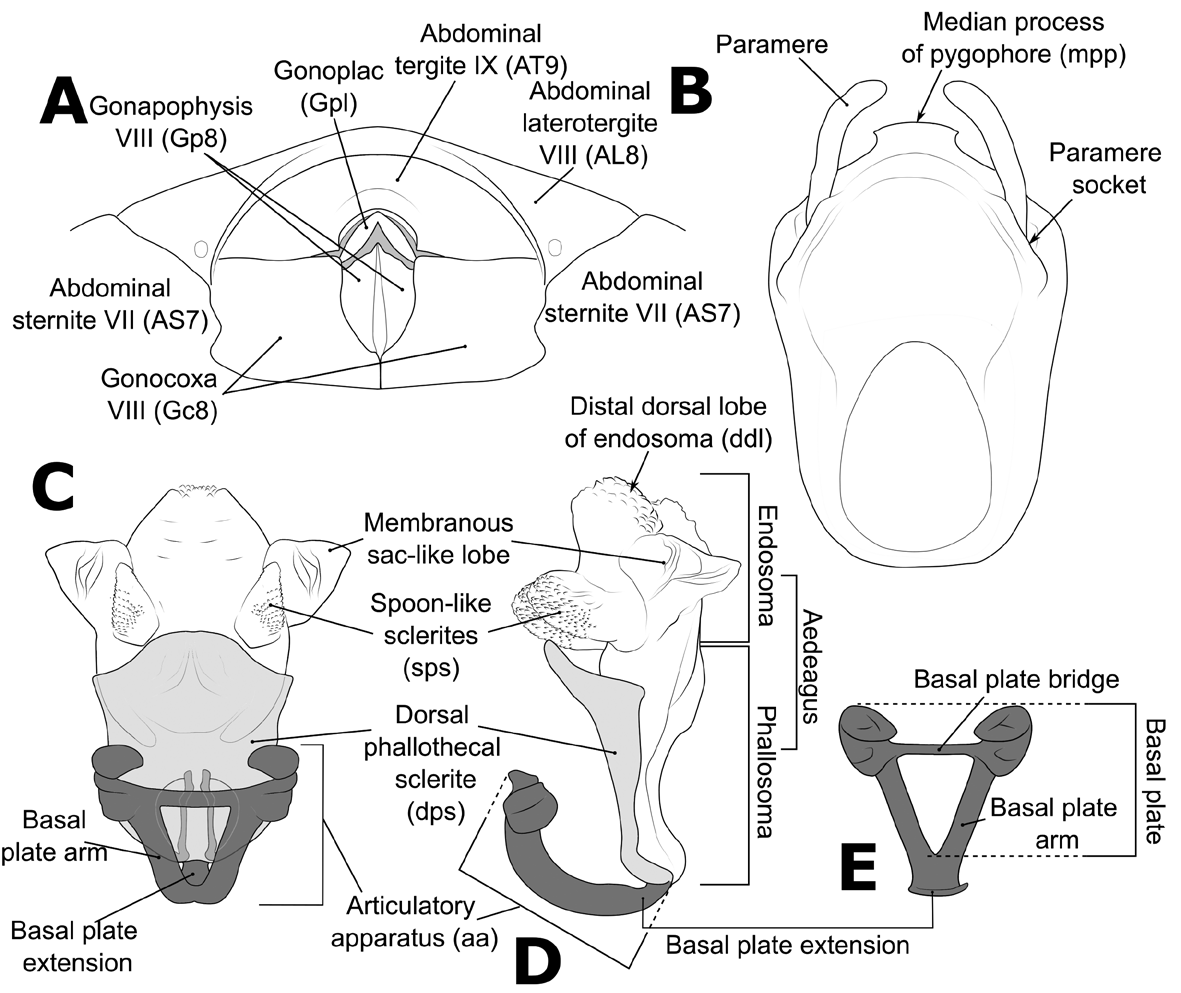
Structure and morphological terms of *Biasticus* species. Drawing based on *Biasticusluteicollis* Ha, Truong & Ishikawa, sp. nov. **A** paratype ♀ HNL2018-024 **B–E** holotype ♂ HNL2018-025. **A** female external genitalia in ventral view **B** pygophore with parameres of male genitalia in dorsal view **C** phallus in dorsal view **D** phallus in lateral view **E** articulatory apparatus in ventral view.

**Figure 4.**
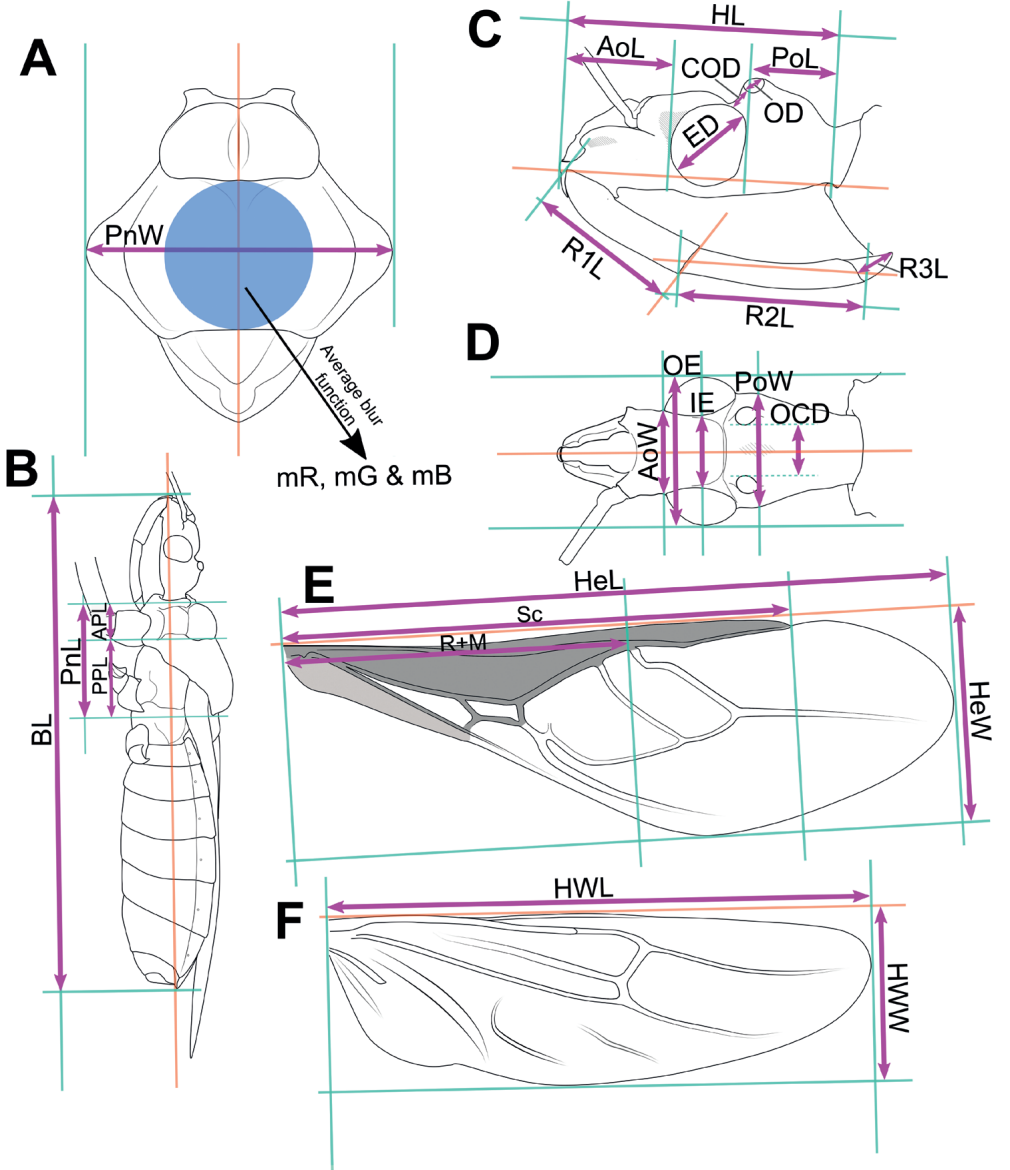
Measurement characters of *Biasticus* species. Drawing based on *Biasticusluteicollis* Ha, Truong & Ishikawa, paratype, sp. nov., ♀, HNL2018-024. **A** pronotum and scutellum in dorsal view, the circle in the center of posterior pronotal lobe with the diameter equal to the minimum length of posterior pronotal lobe, at which the RBG color information was measured for calculating the average RBG color information in the form “mR”, “mG”, “mB” **B** body in lateral view **C** head in lateral view **D** head in dorsal view **E** hemelytron in dorsal view **F** hind wing in dorsal view.

Morphological terminology (Figs 1–3) followed that of Schuh and Weirauch (2020), Forero and Weirauch (2012), and Rosa et al. (2005). The following parts of the bodies were measured for 52 out of 56 *Biasticus* samples (Table 1; Fig. 4), using the software Image-J (http://imageJ.nih.gov/ij/) based on the direct stacking pictures designed as stated above. The assessment features were stated below (Fig. 4), and all dimensions were given in mm:

**BL** body length excluding hemelytra;

**HL** head length;

**AoL** length of anteocular area of head;

**AoW** width of anteocular area of head, measured immediately in front of compound eyes;

**PoL** length of postocular area of head;

**PoW** maximum width of postocular area of head;

**OE** maximum distance measured between outer margins of compound eyes;

**IE** width of synthlipsis, minimum distance measured between inner margins of compound eyes;

**ED** maximum diameter of left compound eye;

**OD** maximum diameter of left ocellus;

**OCD** minimum distance measured between inner margins of lateral ocelli;

**COD** minimum distance between postero-inner margin of left compound eye and antero-outer margin of left lateral ocellus;

**R1L** length of first visible labial segment;

**R2L** length of second visible labial segment;

**R3L** length of third visible labial segment;

**A1L** length of scape;

**A2L** length of pedicel;

**A3L** length of first flagellomere;

**A4L** length of second flagellomere;

**PnL** pronotal length;

**PnW** maximum pronotal width;

**APL** length of anterior pronotal lobe;

**PPL** length of posterior pronotal lobe;

**HeL** length of right hemelytron;

**HeW** maximum width of right hemelytron;

**Sc** length of Sc of right hemelytron;

**R+M** length of R + M of right hemelytron;

**HWL** length of right hind wing;

**HWW** maximum width of right hind wing;

**AFL** length of left fore femur;

**ATL** length of left fore tibia;

**MFL** length of left mid femur;

**MTL** length of left mid tibia;

**PFL** length of left hind femur;

**PTL** length of left hind tibia.

The RGB color values were produced using Adobe Photoshop Elements 10.0 software from a virtual circle in the center of the posterior pronotal lobe with a diameter equal to the anteromedian and posteromedian edges of the posterior pronotal lobe (blue circle in Fig. 4A). The average blur function was then used to calculate mean color values, such as mR (red), mG (green), and mB (blue).

### ﻿Morphometric analyses

Considering the weak to moderate sexual dimorphism of external morphology, the Principal Component Analyses (**PCA**) were performed separately for the female adult and male adult datasets using R software 4.1.2 (R core team 2021). The morphometric dataset comprised 31 morphological features (head length (HL), length of anteocular area of head (AoL), length of postocular area of head (PoL), width of anteocular area of head (AoW), maximum width of postocular area of head (PoW), maximum distance measured between outer margins of compound eyes (OE), width of synthlipsis (IE), maximum diameter of left compound eye (ED), maximum diameter of left ocellus (OD), minimum distance measured between inner margins of lateral ocelli (OCD), minimum distance between postero-inner margin of left compound eye and antero-outer margin of left lateral ocellus (COD), length of first visible labial segment (R1L), length of second visible labial segment (R2L), length of scape (A1L), length of pedicel (A2L), length of first flagellomere (A3L), length of second flagellomere (A4L), length of right hemelytron (HeL), maximum width of right hemelytron (HeW), length of Sc of right hemelytron (Sc), length of R + M of right hemelytron (R+M), length of left fore femur (AFL), length of left fore tibia (ATL), length of left mid femur (MFL), length of left mid tibia (MTL), length of left hind femur (PFL), and length of left hind tibia (PTL)), and proportion of mean values of three color indices (mR, mG, and mB) of the central region of posterior pronotal lobe to sum of mR, mG, and mB (mRr = mR/(mR + mG + mB), mGr = mG/(mR + mG + mB), and mBr = mB/(mR + mG + mB)). The function “fviz_pca_ind” (factoextra package) (Kassambara and Mundt 2020) was used to graph the 2D plot of PCA. To determine the most prominent contributing morphometric characters in each male and female dataset, we used the “get_pca_var” (factoextra package) function to test the contribution of variables to the dimensions of PCA (Kassambara and Mundt 2020).

Raw morphometric datasets and the R-scripts used for the data design and analyses are presented in additional files (Suppl. materials 1–3).

### ﻿Molecular data preparation

DNA was isolated from the right or left hind tibia of each specimen by the Chelex-TE-ProK protocol (Satria et al. 2015). The mitochondrial 16S and COI gene fragments were examined using the primers presented in Table 2. Polymerase chain reaction (PCR) amplification, cycle sequencing reaction, sequencing using ABI PRISM 3130xl (Applied Biosystems), and sequence assembly using ChromasPro 1.7.6 (Technelysium Pty Ltd., Australia) were executed using the methods of Satria et al. (2015), Simon et al. (1994), Cognato and Vogler (2001), Meusnier et al. (2008), Shekhovtsov et al. (2013), Bely and Wray (2004), and Zhang and Weirauch (2013). The PCR thermal situation for the two gene fragments, 16S and COI, comprised of initial denaturation 94 °C (2 min), denaturation 94 °C (30 s), annealing at appropriate annealing temperature (30 s) (Table 2), and extension 72 °C (45 s) for 35 cycles, with final extension at 72 °C (7 min). COI sequences were effectively obtained from 31 out of the 56 *Biasticus* samples and 16S sequences were effectively derived from 45 of the 56 *Biasticus* samples. The PCR thermal cycles for the mini-barcode of COI, i.e., Uni-Minibar, comprised of initial denaturation 95 °C (2 min), denaturation 95 °C (1 min), annealing 46 °C (1 min), and extension 72 °C (30 s) for 5 cycles, then denaturation 95 °C (1 min), annealing 53 °C (1 min), and extension 72 °C (30 s) for 35 cycles, with final extension at 72 °C (5 min). Uni-Minibar sequences were successfully derived from 47 of the 56 *Biasticus* samples.

Test for association was performed using MUSCLE implemented in MEGA X (Kumar et al. 2018) with default setting (Gap Open = -400.00; Gap Extend = 0.00; Cluster Method [Iterations 1,2 and Other iterations] = UPGMA; Min Diag Length [Lambda] = 24) for COI and 16S sequences while including and excluding outgroups (OG+ or OG−): 16S^(OG+)^ (487 bp), and 16S^(OG−)^ (487 bp), COI^(OG−)^ (603 bp), Uni-Minibar^(OG+)^ (174 bp), Uni-Minibar^(OG−)^ (174 bp) datasets. The 16S^(OG+)^ and Uni-Minibar^(OG+)^ datasets were aggregated to produce a concatenated 16S + Uni dataset (661 bp). The FASTA-configured files derived from MEGA X were then converted to NEXUS layout or PHYLIP design, which were suitable input layouts for molecular phylogenetic examination and estimation of genetic distances and species delimitation analysis by ClustalX 2.0.11 (Larkin et al. 2007).

**Table 2.** Primers used for PCR amplification and Sanger sequencing.

Gene	Forward	Reverse	Annealing temperature	Source
16S	16sa: 5’-CGC CTG TTT ATC AAA AAC AT-3’	16sb: 5’-CTC CGG TTT GAA CTC AGA TCA-3’	48 ^o^C	Kessing et al. (1989)
	LR-J-12961 (F): 5’- TTT AAT CCA ACA TCG AGG -3’	LR-J-13417 (R): 5’- CGC CTG TTT AAC AAA AAC AT -3’	47 ^o^C	Simon et al. (1994) Cognato and Vogler (2001)
COI	Uni-Minibar (F1): 5’- TCC ACT AAT CAC AAR GAT ATT GGT AC -3’	Uni-Minibar (R): 5’- GAA AAT CAT AAT GAA GGC ATG AGC -3’	53 ^o^C	Meusnier et al. (2008)
	LCO1490m: 5’-TAC TCA ACA AAT CAC AAA GAT ATT GG-3’	3’ COI-E: 5’-TAT ACT TCT GGG TGT CCG AAG AAT CA-3’	48.5 ^o^C	Shekhovtsov et al. (2013) Bely & Wray (2004)
	COI_Harp_F: 5’-ATT GGA AAT GAY CAA ATY TAT A-3’	COI_Harp_R: 5’-GAD GTA TTA AAR TTW CGR TCW-3’	48.5 ^o^C	Zhang & Weirauch (2013)

### ﻿Molecular phylogenetic analyses

Molecular phylogenetic analyses were done based on the concatenated 16S + Uni dataset since the number of specimens that were successfully obtained 16S gene fragment and Uni-Minibar gene fragment were the highest. The substitution models, K3Pu + F + I + G4, TIM2e + G4, and (K3Pu + F + I + G4, TIM2e + G4), were selected respectively for the 16S^(OG+)^, Uni-Minibar^(OG+)^, and the concatenated 16S + Uni datasets by Model Finder (Kalyaanamoorthy et al. 2017) executed in IQ-TREE 2.1.2 (Minh et al. 2020). Maximum likelihood (**ML**) examinations were then carried out using IQ-TREE 2.1.2 (Chernomor et al. 2016; Minh et al. 2020); bootstrap values (**BP**) were estimated from 1,000 replications. The generalized time-reversible (**GTR**) + Gamma model was chosen for the 16S + Uni dataset using Model Finder (Kalyaanamoorthy et al. 2017) under the Bayesian information criterion. The Bayesian inference (**BI**) evaluations were then executed for the data using MrBayes v. 3.2.6 (Ronquist and Huelsenbeck 2003) with 20,000,000 production and statutory parameter configuration (examining every 500 generations and tuning constraints every 100 generations, with a burn-in of 25%). The effective sampling size (ESS) of each constraint was verified to be > 200 using Tracer 1.7.1 (Rambaut et al. 2018). The nodes were designated as “well supported” when posterior probability (**PP**) ≥ 0.95 and BP ≥ 80.

### ﻿Species delimitation analyses

To create species partitions, two different protocols, i.e., Assemble Species by Automatic Partitioning (**ASAP**) (Puillandre et al. 2021) and Bayesian implementation of the Poisson Tree Processes model (**bPTP**) for species delimitation (Zhang et al. 2013), were used with pairwise genetic distances. For ASAP, the FASTA-configured files of 16S^(OG−)^, Uni-Minibar^(OG−)^, and COI^(OG−)^ datasets were used and executed on the ASAP website (https://bioinfo.mnhn.fr/abi/public/asap), with two replacement samples to estimate the distances, i.e., simple p-distance model and K2P model. The bPTP were executed in the bPTP online server (https://species.h-its.org) based on the NEXUS formatted input files of 16S^(OG−)^, Uni-Minibar^(OG−)^ and COI^(OG−)^ datasets, with default values (100,000 Markov chain Monte Carlo [MCMC] generations, thinning = 100, burn-in = 0.1, and Seed = 123).

## ﻿Results and discussion

### ﻿Examination of genital morphology in the female and male

**Figure 5.**
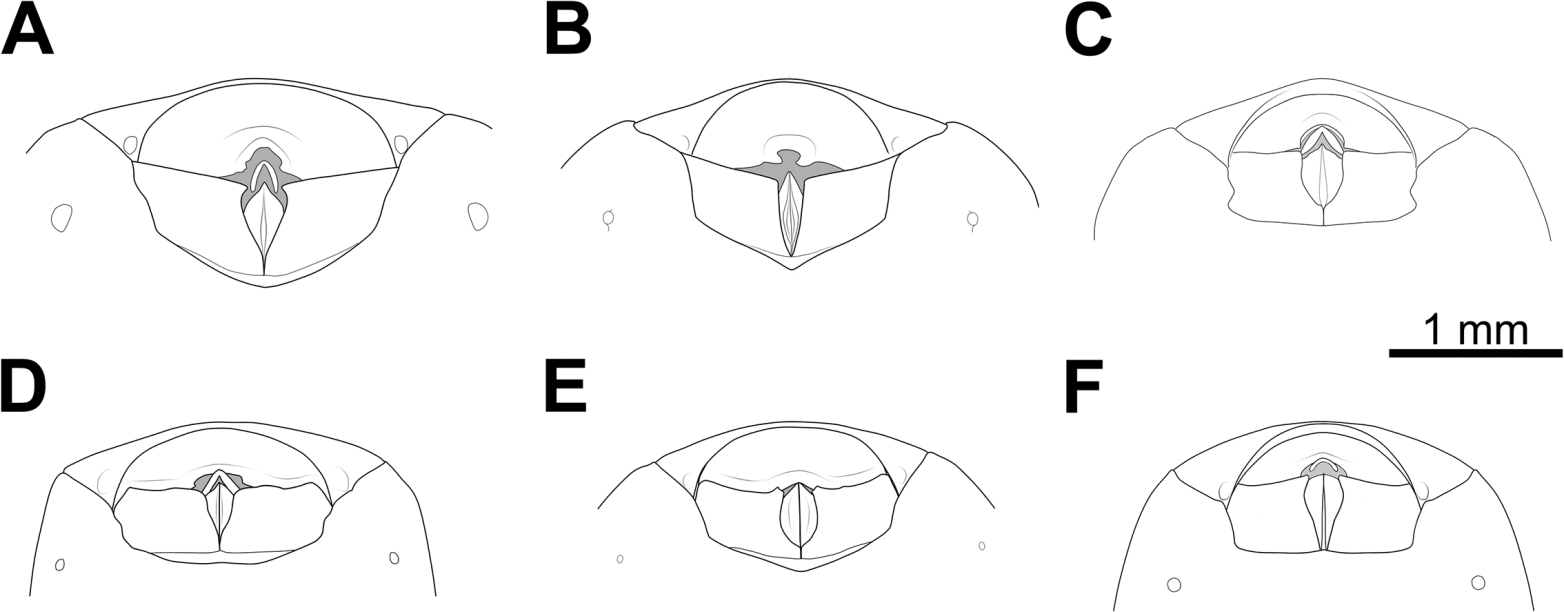
Female external genitalia of six morphospecies in ventral view **A** type fA, *Biasticustaynguyenensis* Ha, Truong & Ishikawa, sp. nov., holotype, ♀, HNL2018-073 **B** type fB, *B.griseocapillus* Ha, Truong & Ishikawa, sp. nov., holotype, ♀, HNL2018-038 **C** type fC, *B.luteicollis* Ha, Truong & Ishikawa, sp. nov., paratype, ♀, HNL2018-024 **D** type fD, *B.flavinotus* (Matsumura, 1913), ♀, HNL2018-117 **E** type fE, *B.confusus* Hsiao et al., 1979, ♀, VN-Hem-1998-012 **F** type fF, *B.flavus* (Distant, 1903), ♀, LA-Redu-2004-006.

Thirty-five female specimens were grouped into six female-based morphospecies (fA–fF) based on the characteristics observed in the externally visible part of genitalia. The type fA (= *Biasticustaynguyenensis* sp. nov.) (6 specimens) was characterized by the following features: abdominal sternite VII (AS7) forming a semicircular or broad subpentagonal median concavity, with inner posterolateral margin very weakly sinuate; gonocoxa VIII (Gc8) with posterior margin gently slanting anteromesad, with apical inner corner weakly produced mesad and forming an acute apex, with inner margin weakly incurved in rear 2/3; abdominal laterotergite VIII (AL8) visible as a thin bridge above the posteromedian part of abdominal tergite IX (AT9) (Figs 5A, 15A). The type fB (= *B.griseocapillus* sp. nov.) (3 specimens) was characterized by the following features: AS7 forming a broad subpentagonal posteromedian concavity, with inner posterolateral margin almost straight; Gc8 with posterior margin gently slanting anteromesad, with an apical inner corner but not formed, with inner margin strongly slanting anteromesad and slightly incurved; AL8 visible as a thin bridge above the posteromedian part of AT9 (Figs 5B, 18A). The type fC (= *B.luteicollis* sp. nov.) (12 specimens) was characterized by the following features: AS7 producing a wide subrectangular concavity, with posteromedian margin almost straight, with inner posterolateral margin poorly sinuous; Gc8 with almost horizontal and poorly sinuous posterior margin, with apical inner corner weakly and formed posteromesad and forming a blunt apex, with inner margin strongly incurved in its posterior 2/3; AL8 visible as a relatively thick bridge above the posteromedian part of AT9 (Figs 5C, 21A). The type fD (= *B.flavinotus*) (7 specimens, including lectotype) was characterized by the following features: AS7 forming a wide subrectangular concavity, with posteromedian margin poorly incurved, with inner posterolateral margin poorly sinuous; Gc8 with almost horizontal and poorly sinuous rear margin, with rounded apical inner corners, with inner margin considerably slanting anteromesad in rear 2/3; AL8 visible as a thin bridge above the posteromedian part of AT9 (Figs 5D, 7F). The type fE (= *B.confusus*) (3 specimens) was characterized by the following features: AS7 forming a wide subpentagonal concavity, with inner posterolateral margin almost straight; Gc8 with rear margin slightly slanting anteromesad and almost incurved, with apical inner corner poorly formed posteromesad and producing a blunt apex, with inner margin considerably incurved in its rear 2/3; AL8 apparent as a thin linkage above the posteromedian part of AT9 (Figs 5E, 8F). The type fF (= *B.flavus*) (5 specimens) was characterized by the following features: AS7 forming a wide subrectangular concavity, with posteromedian margin nearly straight, with inner posterolateral margin almost straight; Gc8 with posterior margin slightly slanting posteromesad and slightly incurved, with apical inner corner poorly developed posteromesad and producing an acute apex, with inner margin weakly sinuous; AL8 visible as a thin bridge above the posteromedian part of AT9 (Figs 5F, 10A).

**Figure 6.**
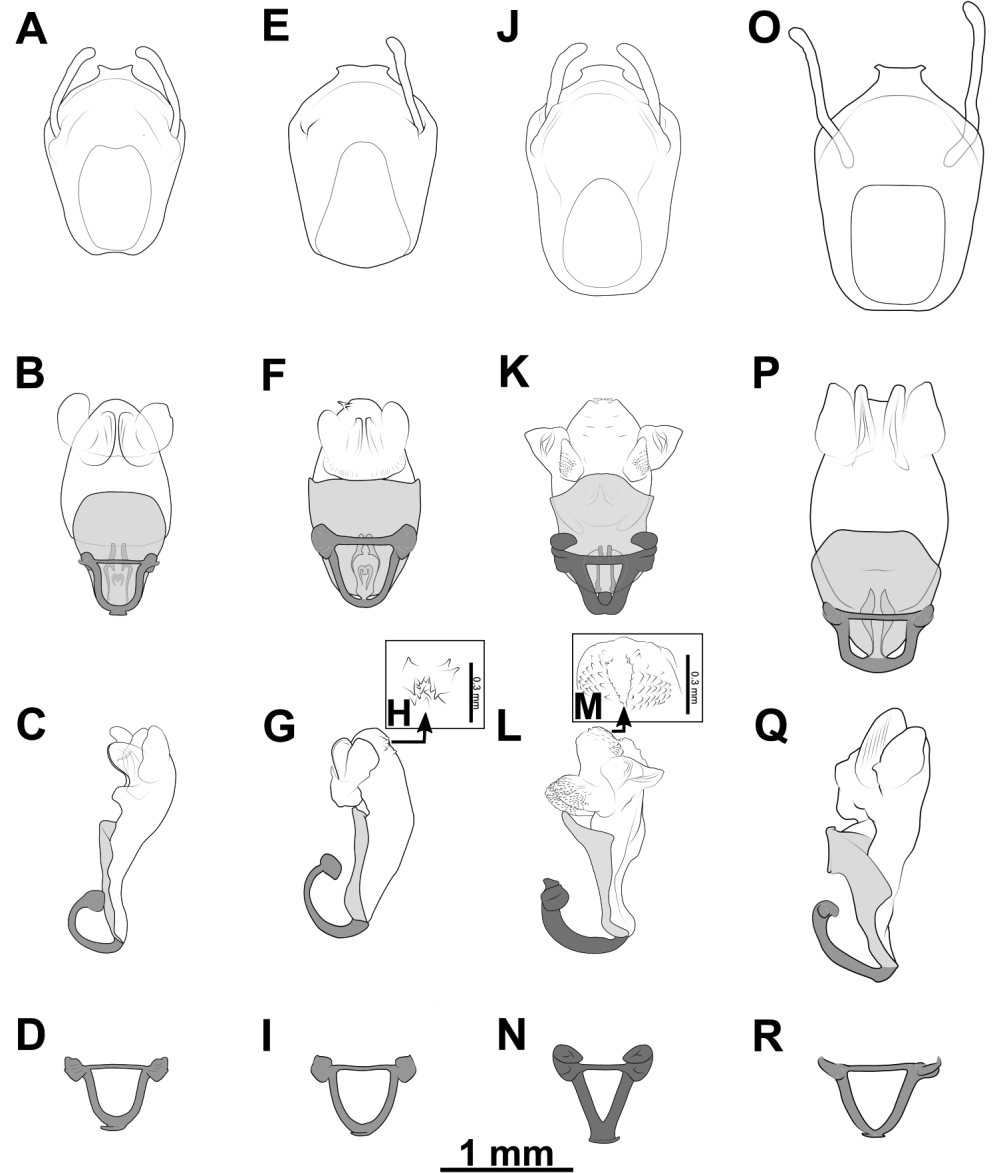
Male genitalia of four morphospecies **A–D** type mA, *Biasticustaynguyenensis* Ha, Truong & Ishikawa, sp. nov., paratype, ♂, TXL2016-545 **E–I** type mB, *B.griseocapillus* Ha, Truong & Ishikawa, sp. nov., paratype, ♂, TXL2016-546 **J–N** type mC, *B.luteicollis* Ha, Truong & Ishikawa, sp. nov., holotype, ♂, HNL2018-025 **O–R** mF, *B.flavus* (Distant, 1903), ♂, HEM-TH2004-018. **A, E, J, O** pygophore with paramere(s) in dorsal view **B, F, K, P** phallus in dorsal view **C, G, L, Q** phallus in lateral view **D, I, N, R** articulatory apparatus **H, M** distal dorsal lobe of endosoma.

In contrast, based on characteristics present in male genitalia (pygophore and aedeagus), 17 adult male specimens were divided into four male-based morphospecies (mA–mC, mF). The type mA (= *B.taynguyenensis* sp. nov.) (1 specimen) was characterized by the following features: the median process of pygophore (mpp) broad and low, with apical margin weakly and progressively concave, with apicolateral corner specifically formed posterolaterad and emarginated (Figs 6A, 15B–E); endosoma with spoon-like sclerites (sps) hyaline and glabrous (Figs 6B, 15I); distal dorsal lobe of endosoma (ddl) round, with membranous surface roughly lumpy; dorsal phallothecal sclerite (dps) in lateral view with posteromedian part poorly formed posterodorsad (Figs 6C, 15K); articulatory apparatus (aa) in ventral view with comparatively slender basal plate arms that mutually form a U-shape (Figs 6D, 15H), in lateral view arched intensely (Figs 6C, 15G). The type mB (*B.griseocapillus* sp. nov.) (1 specimen) was characterized by the following features: mpp broad and low, with apical margin slightly convex and emarginate medially, with apicolateral corner slightly formed laterad and not emarginated (Figs 6E, 18B–E); sps hyaline and glabrous (Fig. 6F, 18J); ddl round, with membranous surface enclosed with large hyaline prickles (Figs 6G, H, 18L, M); dps in lateral view with posteromedian part slightly formed posterodorsad (Figs 6G, 18L); aa in ventral view with comparatively slender basal plate arms that mutually form a U-shape (Figs 6I, 18I), in lateral view arched intensely (Figs 6G, 18H). The type mC (= *B.luteicollis* sp. nov.) (8 specimens) was characterized by the following features: mpp broad and low, with apical margin slightly and constantly convex and did not emarginate, with apicolateral corner slightly formed laterad and emarginated (Figs 6J, 21B–E); sps semi-hyaline and enclosed with tiny blunt spikes (Figs 6K, 21F); ddl round, with a membranous surface covered with small but distinctive spikes (Figs 6L, M, 21G, J); dps in lateral view with posteromedian part very strongly developed posterodorsad (Figs 6L, 21G); aa in ventral view with comparatively broad basal plate arms that mutually formed a V-shape (Figs 6N, 21I), in lateral view arched slightly (Figs 6L, 21G). The type mF (= *B.flavus*) (6 specimens) was characterized by the following features: mpp broad and low, with apical margin weakly and progressively concave, with apicolateral corner specifically formed posterolaterad and blunt at the apex (Figs 6O, 10B–E); sps hyaline and glabrous (Figs 6P, 10I); ddl round, with membranous surface roughly lumpy (Figs 6Q, 10K); dps in lateral view with posteromedian part poorly formed posterodorsad, producing a flat dorsal outline (Figs 6Q, 10K); aa in ventral view with comparatively slender basal plate arms that mutually form a U-shape (Figs 6R, 10H), in lateral view arched poorly (Figs 6Q, 10G, K).

### ﻿Examination of other morphological features in the female and the male

**Figure 7.**
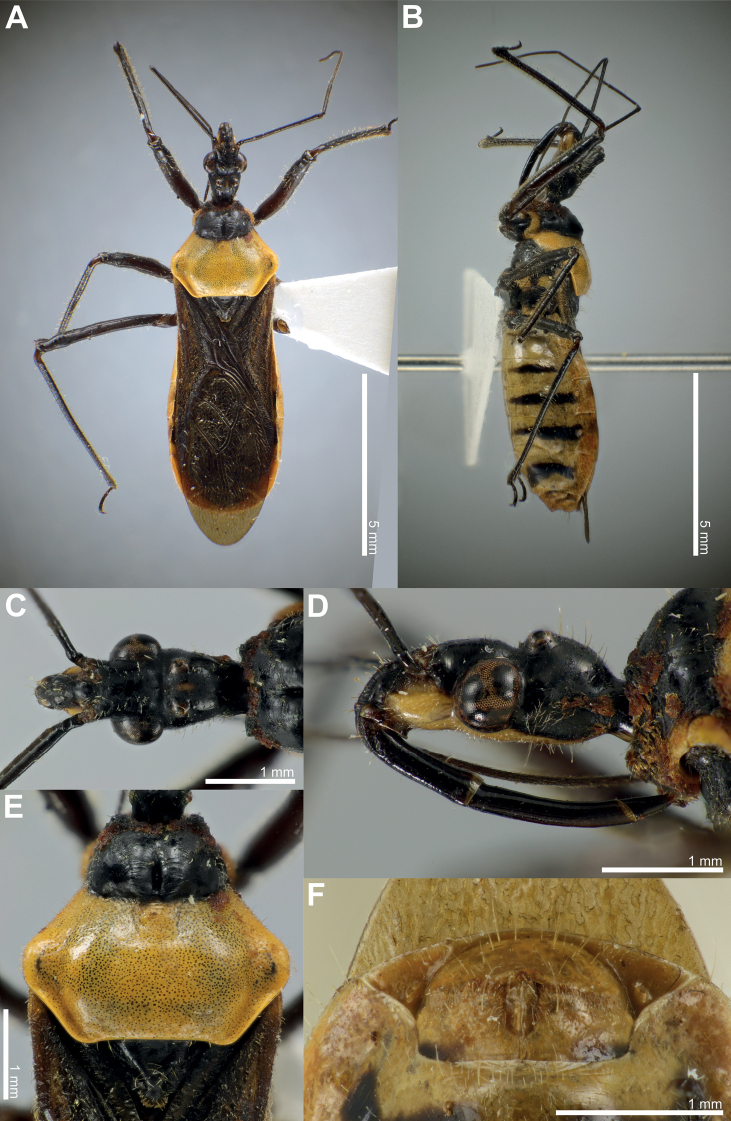
*Biasticusflavinotus* (Matsumura, 1913), ♀, TW-Redu-2014-001 **A** body in dorsal view **B** body in lateral view **C** head in dorsal view **D** head in lateral view **E** pronotum in dorsal view **F** female external genitalia in ventral view.

**Figure 8.**
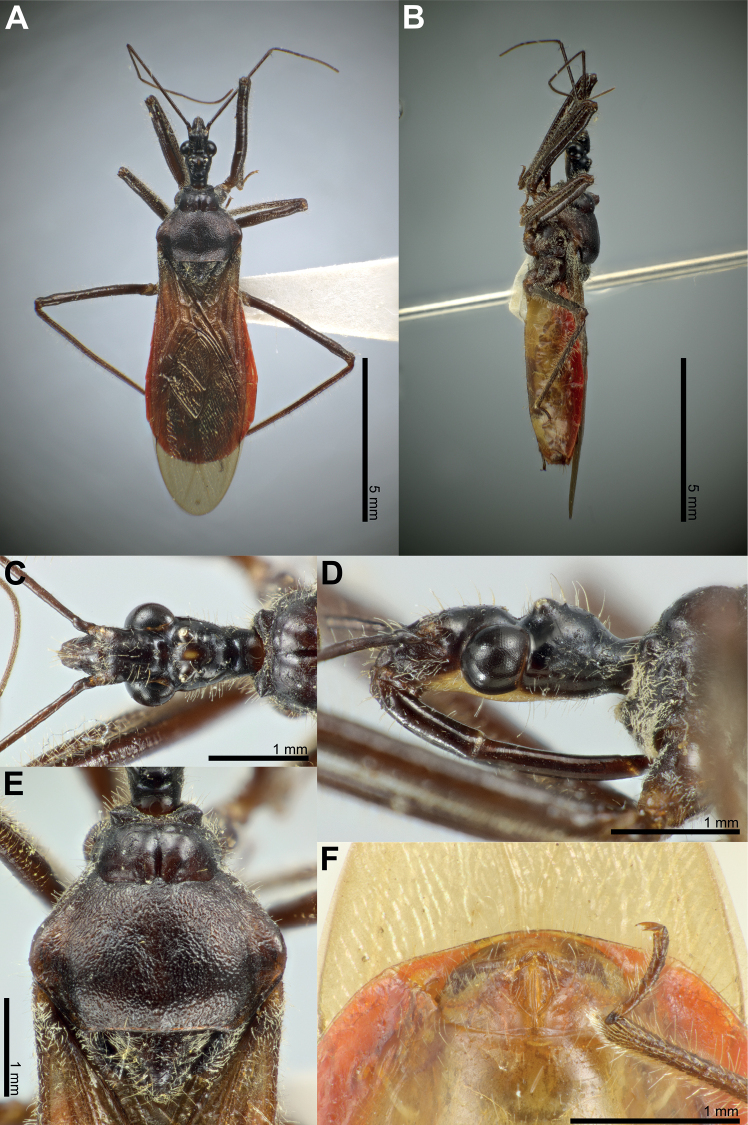
*Biasticusconfusus* Hsiao et al., 1979, ♀, VN-Hem-1998-012 **A** body in dorsal view **B** body in lateral view **C** head in dorsal view **D** head in lateral view **E** pronotum in dorsal view **F** female external genitalia in ventral view.

**Figure 9.**
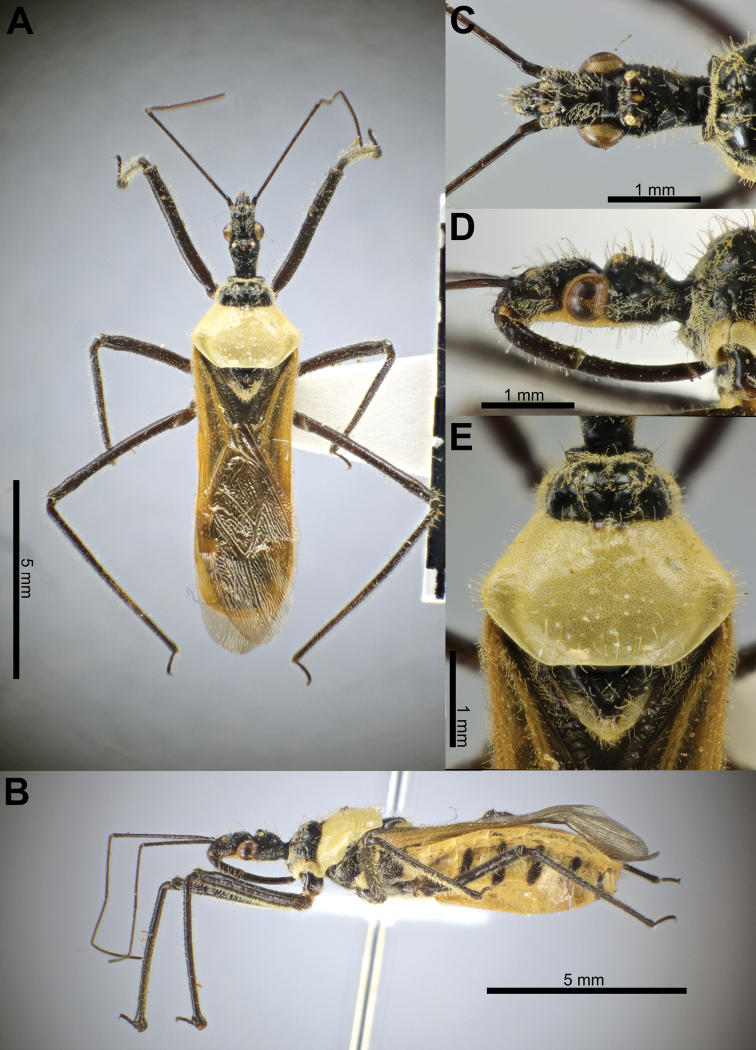
*Biasticusflavus* (Distant, 1903), ♀, LA-Redu-2004-006 **A** body in dorsal view **B** body in lateral view **C** head in dorsal view **D** head in lateral view **E** pronotum in dorsal view.

Fifty-six *Biasticus* specimens were sorted into six morphospecies (bA–bF) based on external body morphology. The type bA (= *B.taynguyenensis* sp. nov.) consisting of fA and mA was characterized by the following features: body shiny blackish brown (Fig. 13A); base of first visible labial segment yellowish brown, remaining of labium black, tips of first and second visible labial segments pale luteous (Fig. 13D); scape ~ 1.5 × as long as head, pedicel slightly longer than first flagellomere and ~ equal in length to second flagellomere; proportional average length of scape, pedicel, first and second flagellomeres 3.1:1.6:1.4:1.6; anterior pronotal lobe blackish brown or black with some rows of short bent cream-yellow setae (Fig. 14A, B); posterior pronotal lobe blackish brown or dark brown, densely covered with short bent cream-yellow setae, interspersed with long erect setae (Fig. 14A, C, D); scutellum wholly black or blackish brown (Fig. 14A); abdominal mediotergites I+II to IV and middle of mediotergite V blackish brown; posterior half of mediotergite V to apex of abdomen, and abdominal sternites sanguineous (Fig. 13A, B); laterotergites II–IV luteous, anterior half of laterotergite V suffused with brown, posterior half of laterotergite V to apex of abdomen sanguineous (Fig. 13B); femora and tibiae blackish brown (Fig. 13A, B). The type bB (= *B.griseocapillus* sp. nov.) consisting of fB and mB was characterized by the following features: body shiny blackish brown (Fig. 16A); first visible labial segment brown, second and third visible labial segments blackish brown, tips of first and second visible labial segments yellowish brown (Fig. 16D); scape ~ 1.5 × as long as head; pedicel slightly longer than first flagellomere and nearly equal in length to second flagellomere; proportional average length of scape, pedicel, first and second flagellomeres 3.0:1.6:1.4:1.6; anterior pronotal lobe black or blackish brown with some rows of long bent griseous setae (Fig. 17A, B); posterior pronotal lobe blackish brown or brown and densely covered with short bent griseous setae somewhat interspersed with long griseous setae (Fig. 17A, C, D); scutellum black in basal half and dark brown or brown in lateral margin and apical half (Fig. 17A); abdominal mediotergites I+II to V reddish brown or sanguineous, somewhat suffused with blackish brown, mediotergite VI suffused with reddish brown and irregularly suffused with sanguineous, mediotergite VII to posterior apex of abdomen sanguineous; abdominal sternites shiny sanguineous (Fig. 16B); laterotergites II–VI luteous, segmentally suffused with dark brown spots or blackish brown spots, laterotergite VII sanguineous (Fig. 16B); femora and tibiae blackish brown. The type bC (= *B.luteicollis* sp. nov.) consisting of fC and mC was characterized by the following characters: body shiny luteous (Fig. 19A); first visible labial segment and base of second visible labial segment luteous, apical 2/3 of second visible labial segment to third visible labial segment yellowish brown or brown (Fig. 19D); scape ~ 1.3 × as long as head; pedicel ~ as long as first flagellomere and ~ 0.7 × as long as second flagellomere; proportional average length of scape, pedicel, first and second flagellomeres 2.7:1.3:1.3:1.7; pronotum covered with long thick erect setae (Fig. 20A, C); anterior pronotal lobe, except lateral margin, shiny dark brown, sometimes with luteous suffusion centrally (Fig. 20B); posterior pronotal lobe luteous (Fig. 20A); scutellum dark brown in basal half and luteous in apical half, with median pale brownish luteous portion (Fig. 20A); abdominal mediotergites, except posterior half of mediotergite VII, dark brown and darker backward, posterior half of mediotergite VII luteous; abdominal sternites pale luteous (Fig. 19B); connexivum pale luteous, with segmentally dark brown suffusions (Fig. 19B); femora luteous with dark brown or yellowish brown suffusions at apex and sometimes at middle (Fig. 19A). The type bD consisting of fD only was characterized by the following features: body black (Fig. 7A); first and third visible labial segments and base of second visible labial segment black, remaining of second visible labial segment blackish brown (Fig. 7D); scape nearly 1.5 × as long as head; pedicel ~ 0.6 × as long as first flagellomere and ~ 0.4 × as long as second flagellomere; proportional average length of scape, pedicel, first and second flagellomeres 2.9:1.1:1.7:2.4; pronotum covered with short bent cream-yellow setae (Fig. 7E); anterior pronotal lobe black, posterior pronotal lobe luteous (Fig. 7E); scutellum wholly blackish brown to black (Fig. 7E); abdominal mediotergites, except lateral margins of mediotergite VII, blackish brown or dark reddish brown, lateral margins of mediotergite VII luteous; abdominal sternites luteous with some blackish brown or black segmental transverse stripes laterally (Fig. 7B); connexivum yellow to sanguineous (Fig. 7B); femora and tibiae black (Fig. 7A). This morphospecies corresponded to *B.flavinotus*. The type bE consisting of fE only was characterized by the following features: body shiny black (Fig. 8A); labium dark brown (Fig. 8D); scape ~ 1.7 × as long as head, pedicel approximately equal in length to first flagellomere and shorter than second flagellomere; proportional average length of scape, pedicel, first and second flagellomeres 3.5:1.6:1.6:2.1; pronotum dark brown to blackish brown (Fig. 8E); central disc of scutellum blackish brown, remaining of scutellum dark brown (Fig. 8E); abdominal mediotergites and sternites luteous to pale sanguineous (Fig. 8B); connexivum sanguineous (Fig. 8B); femora and tibiae dark brown to blackish brown (Fig. 8A). This morphospecies corresponded to *B.confusus*. The type bF consisting of fF and mF was characterized by the following features: body shiny black (Fig. 9A); labium blackish brown, paler apically (Fig. 9D); scape ~ 1.5 × as long as head, pedicel slightly longer than first flagellomere and subequal in length to second flagellomere; proportional average length of scape, pedicel, first and second flagellomeres 3.2:1.7:1.4:1.7; pronotum densely covered with long thick yellow erect setae (Fig. 9E); anterior pronotal lobe blackish brown to black with some rows of short yellow bent setae (Fig. 9E); posterior pronotal lobe luteous, somewhat anteriorly centrally suffused with blackish brown (Fig. 9E); scutellum blackish brown to black, except posterior halves of lateral margins and posterior apex luteous (Fig. 9E); abdominal mediotergites luteous with irregular brown suffusion, sometimes mediotergites wholly brown; abdominal sternites luteous with some blackish brown or black segmental transverse stripes laterally (Fig. 9B); connexivum pale luteous to luteous (Fig. 9B); femora and tibiae blackish brown to black (Fig. 9A). This morphospecies corresponded to *B.flavus*.

### ﻿Morphometric analyses

The six female-based morphospecies (fA–fF) were discriminated from each other by PCA based on the morphometric dataset of female adults (Fig. 11A). Similarly, PCA based on the morphometric dataset of male adults, also revealed a significant separation of two groups, i.e., mC and *B.flavus* (mF) and three singletons, i.e., mA, mB, and *B.confusus* (mE) (Fig. 11B). For *B.flavinotus*, the mature male specimen was unavailable.

### ﻿Phylogenetic analyses and DNA barcoding

Based on the concatenated 16S + Uni dataset (Fig. 12), each of the six morphospecies (bA–bF) was recovered as an independent clade with high supporting values (PP = 1; BP ≥ 93) and long basal branches in both BI and ML trees. The phylogenetic portioning was supported consistently by ASAP and bPTP based on the COI^(OG−)^, Uni-Minibar^(OG−)^, and 16S^(OG−)^ datasets (Fig. 12).

That is to say that the conspecific female and male association was presumed for the following cases: bA (fA = mA), bB (fB = mB), bC (fC = mC) and bF (fF = mF; *B.flavus*). The males of *B.flavinotus* and *B.confusus* have not yet been collected by us.

### ﻿Species discrimination and identification

The bA, bB, and bC, of which each was confirmed to be an independent species, were distinguished also from 20 named congeners including the following three species already known from Vietnam, namely, *B.confusus* Hsiao et al., 1979 (= bE), *B.flavinotus* (Matsumura, 1913) (= bD), and *B.flavus* (Distant, 1903) (= bF), based on the features of external and genital morphology that have been used in diagnosing species of *Biasticus* and other related genera. The species bA, bB, and bC are therefore named and described as *Biasticustaynguyenensis* Ha, Truong & Ishikawa, sp. nov., *B.griseocapillus* Ha, Truong & Ishikawa, sp. nov., and *B.luteicollis* Ha, Truong & Ishikawa, sp. nov., respectively.

The present study effectively identified a set of morphological features in female and male adults that can be used to classify aged specimens in existing collections that are not suitable for molecular phylogenetic analysis: the morphology of the posterior margin of AS7 and of the apical inner corner and the posterior and inner margins of Gc8 in the female genitalia; the morphology of mpp, sps, ddl, and dps and aa in the male genitalia; length of scape (A1L), length of pedicel (A2L), length of second flagellomere (A4L), length of right hemelytron (HeL), length of Sc in the right hemelytron (Sc), proportions of mean values of green and blue color indicators (mG and mB) of the central region of rear pronotal lobe to sum of mR, mG, and mB (mGr = mG/(mR + mG + mB), and mBr = mB/(mR + mG + mB)), head length (HL), and length of anteocular area of head (AoL) of the female adult (Table 3); length of left fore and hind tibiae (ATL, PTL), length of left femora (AFL, MFL, PFL), proportion of mean value of green color indicators (mG) of the central region of rear pronotal lobe to sum of mR, mG, and mB (mGr = mG/(mR + mG + mB)), length of scape (A1L), and maximum diameter of left ocellus (OD) of the male adult (Table 3).

### ﻿Taxonomic account

#### Family Reduviidae Latreille, 1807

**Subfamily Harpactorinae Amyot & Serville, 1843**

##### Genus *Biasticus* Stål, 1867

###### ﻿ Biasticus taynguyenensis

Taxon classificationAnimaliaHemipteraReduviidae

Ha, Truong & Ishikawa sp. nov.

3AE772A8-41AA-5A6B-BEA5-FBE68166A037

https://zoobank.org/290765E6-63AE-49F4-B835-B22A034E4DE7

Figs 5A , 6A–D , 13 , 14 , 15

####### Type material.

***Holotype*.** ♀; HNL2018-073; Vietnam, Gia Lai Province, Kon Chu Rang Nature Reserve; 08.v.2018; X. L. Truong leg.; NSMT. ***Paratypes*.** 1♀; HNL2018-036; Vietnam, Dak Lak Province, Chu Yang Sin National Park; 09.v.2018; X. L. Truong leg.; IEBR. 2♀; HNL2018-072; HNL2018-076; Vietnam, Gia Lai Province, Kon Chu Rang Nature Reserve; 08.v.2018; X. L. Truong leg.; IEBR. 1♀; HNL2018-074; Vietnam, Gia Lai Province, Kon Chu Rang Nature Reserve; 08.v.2018; X. L. Truong leg.; NSMT. 1♀; HNL2018-075; Vietnam, Gia Lai Province, Kon Chu Rang Nature Reserve; 08.v.2018; X. L. Truong leg.; VNMN. 1♂; TXL2016-545; Vietnam, Gia Lai Province, Kon Chu Rang Nature Reserve; 28.iv.2016; X. L. Truong leg.; NSMT.

####### Diagnosis.

Body shiny blackish brown; anterior pronotal lobe blackish brown or black with some rows of short bent cream-yellow setae; posterior pronotal lobe blackish brown or dark brown, densely covered with short bent cream-yellow setae, interspersed with long erect setae; scutellum black or blackish brown; abdominal sternites sanguineous; laterotergites II–IV luteous; anterior half of laterotergite V suffused with brown; posterior half of laterotergite V to apex of abdomen sanguineous.

This species is similar to *B.confusus* Hsiao et al., 1979 in general appearance, especially in body color, pronotum, and thoracic sterna. But the new species can be distinguished from *B.confusus* by a combination of the following characters: antennal pedicel longer than first flagellomere (in *B.confusus* pedicel as long as first flagellomere), second flagellomere as long as pedicel (in *B.confusus* second flagellomere longer than pedicel), proportional average length of first to third labial segments 1.1:1.4:0.3 (in *B.confusus* 0.9:1.2:0.3), anterior pronotal lobe with some rows of bent setae (in *B.confusus* without row of bent setae), posterior pronotal lobe ~ 2.3 × as long as anterior pronotal lobe (in *B.confusus* 2.0 ×), and apical margin of median process of pygophore weakly and continuously concave (in *B.confusus* weakly and continuously convex).

Furthermore, this species is somewhat similar to *B.ventralis* Hsiao et al., 1979 in general colors of body, pronotum, and sterna but the new species can be distinguished from *B.ventralis* by a combination of the following characters: antennal pedicel longer than first flagellomere (in *B.ventralis* pedicel ~ 1/2 as long as first flagellomere), second flagellomere as long as pedicel (in *B.ventralis* second flagellomere 2.5 × as long as pedicel), proportional average length of antennal scape, pedicel, first and second flagellomeres 3.1:1.6:1.4:1.6 (in *B.ventralis* 3.3:1.1:2.0:2.8), and proportional average length of first to third labial segments 1.1:1.4:0.3 (in *B.ventralis* 1.0:1.3:0.3).

####### Description.

**Female description. *Coloration*.** Body shiny blackish brown. Head dorsum shiny black or blackish brown; clypeus blackish brown; antenniferous tubercle, base of neck, maxillary plate, and base of first visible labial segment yellowish brown; central fascia to head venter, gena, tips of first and second visible labial segments pale luteous; labium, except base of first visible labial segment, black; a brown stripe present after postero-upper corner of compound eye; area around lateral ocellus with reddish brown suffusion; a longitudinally elongated yellowish brown spot present between lateral ocelli. Base of scape dark brown; remaining of scape brown; pedicel, first and second flagellomeres brown, darker toward tip. Collar, anterior pronotal lobe, thoracic sterna, and pleura blackish brown or black; posterior pronotal lobe blackish brown; scutellum black or blackish brown; stridulatory sulcus, intersecting area of coxa and trochanter yellowish brown; coxae, trochanters, femora, and tibiae blackish brown. Corium and clavus blackish brown, apically brownish yellow; membrane bronzy brown, semi-hyaline. Hind wings faintly semi-hyaline. Abdominal mediotergites I+II to IV and middle of mediotergite V blackish brown; posterior half of mediotergite V to apex of abdomen and abdominal sternites sanguineous; laterotergites II–IV luteous; anterior half of laterotergite V suffused with brown; posterior half of laterotergite V to apex of abdomen sanguineous. Female external genitalia sanguineous.

***Structure*.** Body medium-sized (BL = 10.38–11.35 mm), elongate, and somewhat robust. Head subelongate and robust (HL/PoW = 2.40–2.59), shorter than pronotum (HL/PnL = 0.80–0.86); postocular area of head sub-globose (PoL/PoW = 0.84–0.96), distinctly wider than anteocular area (PoW/AoW = 1.38–1.43), approximately as long as anteocular area (PoL/AoL = 0.95–1.08), constricted behind compound eyes, with a wide and deep interocular sulcus; neck short. Compound eyes protruding laterally, nearly globose, with posterior margin sub-straight; lateral ocelli produced, elevated behind interocular sulcus, widely separated from each other (OCD/PoW = 0.44–0.49); interspace between lateral ocelli wider than distance between compound eye and lateral ocellus (OCD/COD = 2.03–2.06). First visible labial segment shorter than second segment (R1L/R2L = 0.76–0.84), longer than anteocular area of head (R1L/AoL = 1.42–1.60), extending beyond level of middle of compound eye when labium laid backward; proportional average length of first to third visible labial segments 1.1:1.4:0.3. Scape ~ 1.5 × as long as head; pedicel slightly longer than first flagellomere and nearly equal in length to second flagellomere; proportional average length of scape, pedicel, first and second flagellomeres 3.1:1.6:1.4:1.6. Collar very short in dorsal view, with anterolateral angle weakly and roundly produced; anterior pronotal lobe round and bulged, slightly rough, with middle longitudinal sulcus deep and narrow posteriorly; posterior pronotal lobe with slightly swollen anteromedial elevation; humerus roughly triangular, with round apex; posterior margin of pronotum straight; posterior angles round, not exceeded to posterior margin of pronotum. Scutellum triangular, somewhat triangularly depressed basally, apically produced, and sloping downward; posterior apex round; anterior margin slightly convex. Femora thick, apically moderately nodulose; fore femora very slightly incrassated, thicker than mid and hind femora. Hemelytra surpassing apex of abdomen when fully closed, 0.8 × as long as body length; discal cell nearly parallelogram-shaped, twice as long as width; Sc 0.8 × as long as hemelytron length, 1.5 × as long as R + M. Hind wing ~ 3.4 × as long as maximum width. Connexivum slightly dilated and ascending with segmental incisures; abdominal laterotergite VIII (AL8) with thin posterior margin (0.03 mm); abdominal sternite VII (AS7) forming a semi-circular or wide sub-pentagonal median concavity, with posteromedian margin gently U-shaped, with inner posterolateral margin almost straight; gonocoxa VIII (Gc8) ~ 1.3 × wider than length, gently slanting anteromesad along posterior margin, weakly produced mesad and forming an acute apex at apical inner corner, and with inner margin weakly incurved in posterior 2/3; gonapophysis (Gp8) small and subtriangular, 3.2 × longer than width; gonoplac (Gpl) V-shaped, thin, with maximum thick ~ 0.025 mm.

***Vestiture*.** Body clothed with cream-yellow setation. Head, except interocular sulcus and area along anterior margin of compound eye, covered with short bent cream-yellow pubescence, and sparsely with long erect setae; labium with a few bent setae; scape without setae and pubescence; pedicel, first and second flagellomeres with short vertical setae; neck glabrous. Collar, anterior margin, lateral area of anterior pronotal lobe, posterior pronotal lobe, scutellum, pleura, thoracic sterna, and coxae densely covered with short bent cream-yellow pubescence; posterior pronotal lobe, pleura, thoracic sterna, and coxae interspersed with long erect setae; scutellum with long bent slender setae, especially in lateral slopes and in posterior apex; trochanters, femora, and tibiae with short erect setae; corium with short bent setae. Abdomen (including connexivum), except segment VII, with short slender vertical setae; gonocoxa VIII (Gc8) with long slender bent setae.

**Male description.** General external morphology similar to that of the female.

***Coloration*.** Almost similar to female but slightly brighter than female. Clypeus dark brown; anteclypeus, base of first visible labial segment pale yellowish brown; first and third visible labial segments brown; second visible labial segment dark brown; area around ocellus with pale brown suffusion. Prosternum and propleuron blackish brown; posterior pronotal lobe dark brown; scutellum blackish brown; meso- and metapleura brown or yellowish brown; meso- and metasterna yellowish brown; fore coxa pale yellowish brown; coxae and trochanters of mid and hind legs yellowish brown; femora and tibiae dark brown or blackish brown. Pygophore ventrally orangish sanguineous; paramere semi-hyaline, pale orange.

***Structure*.** Almost same as female except for the following characters. Scape 1.7 × as long as head; pedicel, first and second flagellomeres missing. Hemelytra surpassing apex of abdomen when fully closed, nearly 0.9 × as long as body length. Pygophore elliptic; median process of pygophore (mpp) broad and low, 0.3 × as long as wide, with apical margin weakly and continuously concave, with apicolateral corner distinctly produced posterolaterad and pointed; paramere long, slender, clavate, somewhat incurved in apical part, with round apex (Figs 6A, 15B–E). Aedeagus in dorsal view ovoid, dorsally sclerotized (Figs 6B, 15F, I) and in lateral view long and narrow (Figs 6C, 15G, K); articulatory apparatus (aa) in ventral view with basal plate arms relatively slender and jointly forming a U-shape, and in lateral view arched very strongly (Figs 6C, D, 15G, H); dorsal phallothecal sclerite (dps) in lateral view with posteromedian part weakly produced posterodorsad (Figs 6C, 15G, K); spoon-like sclerites (sps) hyaline and glabrous; both membranous sac-like lobes posterolaterally produced; distal dorsal lobe of endosoma (ddl) round, with membranous surface roughly lumpy (Fig. 15L).

***Vestiture*.** Almost same as female except for the following characters. Head covered with short bent cream-yellow pubescence, and sparsely with long bent setae; pedicel with short vertical setae; first and second flagellomeres missing; neck without setae. Scutellum densely covered with short bent cream-yellow pubescence interspersed with long erect setae; trochanters, femora, and tibiae with long erect setae; connexivum with long vertical thick setae; abdominal venter with vertical setae; pygophore with oblique setae; paramere with long erect setae.

####### Measurements.

All dimensions are given in mm. ***Holotype*** (♀): BL 11.35; HL 2.12; AoL 0.76; AoW 0.58; PoL 0.76; PoW 0.82; OE 1.11; IE 0.57; ED 0.65; OD 0.17; OCD 0.41; COD 0.19; R1L 1.22; R2L 1.45; R3L 0.33; A1L 3.13; A2L 1.67; A3L 1.55; A4L n/a; PnL 2.52; PnW 3.01; APL 0.72; PPL 1.80; HeL 8.61; HeW 2.97; Sc 6.59; R+M 4.43; HWL 6.06; HWW 1.84; AFL 3.32; ATL 4.02; MFL 2.76; MTL 3.41; PFL 4.02; PTL 5.53. ***Paratype*** (♂): BL 9.86; HL 1.98; AoL 0.68; AoW 0.56; PoL 0.78; PoW 0.79; OE 1.03; IE 0.54; ED 0.61; OD 0.14; OCD 0.38; COD 0.21; R1L 1.11; R2L 1.39; R3L 0.31; A1L 3.28; A2L n/a; A3L n/a; A4L n/a; PnL 2.31; PnW 2.60; APL 0.70; PPL 1.60; HeL 8.48; HeW 2.72; Sc 6.56; R+M 4.34; HWL 5.60; HWW 1.70; AFL 3.30; ATL 3.91; MFL 2.73; MTL 3.29; PFL 4.00; PTL 5.45. ***Paratypes*** (♀). BL 10.38–10.81; HL 2.00–2.06; AoL 0.72–0.75; AoW 0.58–0.60; PoL 0.70–0.78; PoW 0.82–0.86; OE 1.06–1.09; IE 0.54–0.58; ED 0.62–0.63; OD 0.15–0.17; OCD 0.37–0.41; COD 0.17–0.19; R1L 1.07–1.13; R2L 1.37–1.42; R3L 0.31–0.32; A1L 2.99–3.13; A2L 1.52–1.60; A3L 1.25–1.52; A4L 1.44–1.74; PnL 2.36–2.51; PnW 2.73–2.87; APL 0.66–0.81; PPL 1.64–1.82; HeL 8.63–8.98; HeW 2.78–3.04; Sc 6.59–6.71; R+M 4.51–4.62; HWL 6.03–6.18; HWW 1.77–1.87; AFL 3.11–3.41; ATL 3.79–3.93; MFL 2.64–2.77; MTL 3.30–3.42; PFL 3.73–4.00; PTL 5.35–5.50.

####### Distribution.

Vietnam, Central Highlands (Gia Lai, Dak Lak).

####### Type locality.

Vietnam, Central Highlands, Gia Lai Province, Kon Chu Rang Nature Reserve.

####### Etymology.

This new species is named after the Tay Nguyen region, the local name of Central Highlands.

###### ﻿ Biasticus griseocapillus

Taxon classificationAnimaliaHemipteraReduviidae

Ha, Truong & Ishikawa sp. nov.

EFED6E08-BFEC-5FD1-8C23-8DAFB14CF4EA

https://zoobank.org/BDAB0B60-64D5-4529-8715-2E1B010AD57B

Figs 5B , 6E–I , 16 , 17 , 18

####### Type material.

***Holotype*.** 1♀; HNL2018-038; Vietnam, Dak Lak Province, Chu Yang Sin National Park; 09.v.2018; X. L. Truong leg.; NSMT. ***Paratypes*.** 1♀; HNL2018-007; Vietnam, Gia Lai Province, Kon Chu Rang Nature Reserve; 05.v.2018; X. L. Truong leg.; IEBR. 1♀; HNL2018-037; Vietnam, Dak Lak Province, Chu Yang Sin National Park; 09.v.2018; X. L. Truong leg.; VNMN. 1♂; TXL2016-546; Vietnam, Gia Lai Province, Kon Chu Rang Nature Reserve; 28.iv.2016; X. L. Truong leg.; NSMT.

####### Diagnosis.

Body shiny blackish brown; anterior pronotal lobe black or blackish brown with some rows of long bent griseous setae; posterior pronotal lobe blackish brown or brown and densely covered with short bent griseous setae somewhat interspersed with long griseous setae; scutellum black in basal half and dark brown or brown in lateral margin and apical half; abdominal sternites shiny sanguineous; laterotergites II–VI luteous, segmentally suffused with dark brown spots or blackish brown spots; laterotergite VII sanguineous.

In general appearance, *Biasticusgriseocapillus* sp. nov. resembles *B.confusus* Hsiao et al., 1979, especially in general colors of body, pronotum, and sterna. But the new species can be distinguished from *B.confusus* by a combination of the following characters: antennal pedicel longer than first flagellomere (in *B.confusus* pedicel as long as first flagellomere), second flagellomere of antenna as long as pedicel (in *B.confusus* second flagellomere longer than pedicel), proportional average length of first to third visible labial segments 1.1:1.4:0.3 (in *B.confusus* 0.9:1.2:0.3), anterior pronotal lobe with some rows of bent setae (in *B.confusus* without row of bent setae), posterior pronotal lobe 2.6 × as long as anterior pronotal lobe (in *B.confusus* 2.0 ×), and apical margin of median process of pygophore weakly convex and emarginate at middle (in *B.confusus* weakly convex without such emargination).

Furthermore, this species is similar to *B.ventralis* Hsiao et al., 1979 in general colors of body, pronotum, and sterna but the new species can be distinguished from *B.ventralis* by a combination of the following characters: antennal pedicel longer than first flagellomere (in *B.ventralis* pedicel ~ 1/2 as long as first flagellomere), second flagellomere as long as pedicel (in *B.ventralis* second flagellomere 2.5 × as long as pedicel), proportional average length of antennal scape, pedicel, first and second flagellomeres 3.0:1.6:1.4:1.6 (in *B.ventralis* 3.3:1.1:2.0:2.8), and proportional average length of first to third visible labial segments 1.1:1.4:0.3 (in *B.ventralis* 1.0:1.3:0.3).

Also, *Biasticusgriseocapillus* sp. nov. is similar to *B.taynguyenensis* sp. nov. in the body color pattern, the proportion average length of the antenna segments, and the proportion average length of the visible labial segments. However, *B.griseocapillus* can be distinguished from the latter by a combination of the following characters: collar, anterior pronotal lobe, and posterior pronotal lobe covered with long bent griseous setae (in *B.taynguyenensis* short bent cream-yellow setae), and median process of pygophore (mpp) 0.2 × as long as wide, with apical margin weakly convex and emarginate medially, and with apicolateral corner slightly produced laterad and pointed (in *B.taynguyenensis*, 0.3 × as long as wide, with apical margin weakly and continuously concave, and with apicolateral corner distinctly produced posterolaterad and pointed).

####### Description.

**Female description. *Coloration*.** Body shiny blackish brown. Head dorsum (except anteclypeus, maxillary plate, and gena) black; anteclypeus, labrum, maxillary plate, and anterior region of gena brown; central fascia to head venter, and posterior region of gena pale luteous; first visible labial segment brown; second and third visible labial segments blackish brown; tips of first and second visible labial segments yellowish brown; area around lateral ocellus with reddish brown suffusion; a longitudinally elongated yellowish brown spot present between lateral ocelli. Base of scape brownish black; remaining of scape and pedicel, first and second flagellomeres blackish brown. Collar, anterolateral angles, anterior pronotal lobe, and anterior acetabulum black; posterior pronotal lobe, pleura, and thoracic sterna blackish brown; scutellum black in basal half and dark brown in lateral margin and apical half; stridulatory sulcus luteous; coxae and trochanters yellowish brown; intersecting area of fore coxae and fore trochanters luteous; femora, tibiae, and tarsi blackish brown. Corium and clavus darkish brown, apically brownish yellow; membrane bronzy brown, semi-hyaline. Hind wings faintly semi-hyaline. Abdominal mediotergites I+II to V reddish brown or sanguineous, somewhat suffused with blackish brown; mediotergite VI suffused with reddish brown and irregularly suffused with sanguineous; mediotergite VII and laterotergite VII to apex of abdomen sanguineous; abdominal sternite shiny sanguineous; laterotergites II–VI luteous, segmentally suffused with dark brown spots; laterotergite VII sanguineous. External genitalia sanguineous.

***Structure*.** Body medium-sized (BL = 10.61–10.84 mm), elongate, and somewhat robust. Head subelongate and robust (HL/PoW = 2.37–2.45), shorter than pronotum (HL/PnL = 0.81–0.82); postocular area of head sub-globose (PoL/PoW = 0.85–1.03), distinctly wider than anteocular area (PoW/AoW = 1.44–1.47), slightly longer than anteocular area (PoL/AoL = 1.05–1.27), constricted behind compound eyes, with a wide and deep interocular sulcus; neck short. Compound eyes protruding laterally, nearly globose, with posterior margin sub-straight; lateral ocelli produced, elevated behind interocular sulcus, widely separated from each other (OCD/PoW = 0.43–0.46); interspace between lateral ocelli wider than distance between compound eye and lateral ocellus (OCD/COD = 1.93–2.17). First visible labial segment shorter than second segment (R1L/R2L = 0.80–0.84), longer than anteocular area of head (R1L/AoL = 1.58–1.63), extending beyond level of middle of compound eye when labium laid backward; proportional average length of first to third visible labial segments 1.1:1.3:0.3. Scape ~ 1.5 × as long as head; pedicel slightly longer than first flagellomere and nearly equal in length to second flagellomere; proportional average length of scape, pedicel, first and second flagellomeres 3.0:1.6:1.4:1.6. Collar very short in dorsal view, with anterolateral angle weakly and roundly produced; anterior pronotal lobe round and bulged, slightly rough, with middle longitudinal sulcus deep and narrow posteriorly; posterior pronotal lobe with slightly swollen anteromedial elevation; humerus roughly triangular, with round apex; posterior margin of pronotum straight; posterior angles round, not exceeded to posterior margin of pronotum. Scutellum triangular, somewhat triangularly depressed basally, apically produced and sloping downward; posterior apex round; anterior margin slightly convex. Femora thick, apically moderately nodulose; fore femora very slightly incrassated, thicker than mid and hind femora. Hemelytra surpassing apex of abdomen when fully closed, 0.8 × as long as body length; discal cell nearly parallelogram-shaped, twice as long as width; Sc 0.7 × as long as hemelytron length, 1.4 × as long as R + M. Hind wing ~ 3.2 × as long as maximum width. Connexivum slightly dilated and ascending with segmental incisures; abdominal laterotergite VIII (AL8) with thin posterior margin (0.01 mm); abdominal sternite VII (AS7) forming a wide subpentagonal posteromedian concavity, with inner posterolateral margin almost straight; gonocoxa VIII (Gc8) broad, ~ 1.4 × wider than length, slightly opened inward for gonapophyses VIII (Gp8), with posterior margin gently slanting anteromesad, and with apical inner corner not produced; Gp8 small and subtriangular, 3.9 × longer than width; gonoplac (Gpl) V-shaped, thin, with maximum thick ~ 0.025 mm.

***Vestiture*.** Body clothed with griseous setation. Head, except interocular sulcus and posterior margin of clypeus, covered with long erect setae; posterior margin of clypeus, head venter, anterolateral area of neck with short bent setae and sparsely with long erect setae; anteclypeus, labium, scape with a few short setae; pedicel, first and second flagellomeres covered with short erect to recumbent setae; dorsum of neck without setae. Collar, anterior margin of anterior pronotal lobe with dense bent pubescence; anterior pronotal lobe with some rows of long bent griseous setae; lateral area of anterior pronotal lobe with long erect setae; posterior pronotal lobe densely covered with short bent griseous setae somewhat interspersed with long griseous setae; lateral slope area and posterior apex of scutellum with dense short to long setae; posterior area of posterior pronotal lobe with long erect setae; pleura, thoracic sterna, and coxae with short bent setae; trochanters, femora, and tibiae with long erect setae; corium with bent griseous pubescence. Anterior margin of abdominal mediotergite I+II with some long slender erect yellowish brown setae; mediotergites and dorsal laterotergites sparsely covered with a few short vertical setae; abdominal sternites interspersed with long vertical setae; posterior margin of abdominal laterotergite VIII (AL8) with long erect slender setae; gonocoxa VIII (Gc8) with long slender bent setae; apex of external genitalia with long thick erect setae.

**Male description.** General external morphology similar to that of the female.

***Coloration*.** Almost similar to female but brighter than female. Gena brown in anterior half and luteous in posterior half; labium blackish brown; tips of first and second visible labial segments luteous. Collar, anterolateral angles, anterior pronotal lobe, and anterior acetabulum blackish brown; posterior pronotal lobe, pleura, and thoracic sterna brown; scutellum black in basal half and brown in lateral margin and apical half; stridulatory sulcus pale gray. Laterotergites II–VI pale luteous, segmentally suffused with blackish brown spots; laterotergite VII sanguineous. Pygophore ventrally orpiment-orange; paramere semi-hyaline, pale orange.

***Structure*.** Almost similar to female except for the following characters. Scape 1.8 × as long as head and more than twice as long as pedicel; pedicel slightly longer than first flagellomere; second flagellomere missing; proportional length of scape, pedicel, and first flagellomere 3.5:1.6:1.4. Hemelytra surpassing apex of abdomen when fully closed, 0.9 × as long as body; Sc 0.8 × as long as hemelytron, and 1.5 × as long as R + M. Pygophore ovoid; median process of pygophore (mpp) broad and low, 0.2 × as long as wide, with apical margin weakly convex and emarginate medially, with apicolateral corner slightly produced laterad and pointed; paramere long, slender, clavate, somewhat curved medially, and apically subnodulose and round (Figs 6E, 18B–F). Aedeagus in dorsal view ovoid, dorsally sclerotized (Figs 6F, 18G, J) and in lateral view long and narrow (Figs 6G, 18H, L); articulatory apparatus (aa) in ventral view with basal plate arms relatively slender and jointly forming a U-shape, and in lateral view arched strongly (Figs 6G, I, 18H, I); dorsal phallothecal sclerite (dps) in lateral view with posteromedian part weakly produced posterodorsad (Figs 6G, 18L); spoon-like sclerites (sps) hyaline and glabrous; both membranous sac-like lobes posterolaterally produced; distal dorsal lobe of endosoma (ddl) round, with membranous surface covered with large hyaline prickles (Figs 6H, 18M).

***Vestiture*.** Almost similar to female except for the following characters. Posterior margin of clypeus with short bent pubescence; anteclypeus and scape without setae. Collar and anterior margin of anterior pronotal lobe with short bent setae and some long bent setae; anterior pronotal lobe with some rows of long bent griseous setae; posterior pronotal lobe with bent griseous setae; scutellum with long slender erect setae abundantly, especially in lateral slopes; posterior apex of scutellum with a pinch of long slender erect and bent setae; pleura, thoracic sterna, and coxae with short bent setae, with a few long slender setae; trochanters sparsely with short bent pubescence and a few long setae; corium with bent pubescence. Pygophore ventrally covered with a few bent pubescence, more abundant laterally; paramere with a few long erect thick setae.

####### Measurements.

All dimensions are given in mm. ***Holotype*** (♀): BL 10.84; HL 1.99; AoL 0.67; AoW 0.56; PoL 0.85; PoW 0.82; OE 1.05; IE 0.56; ED 0.60; OD 0.17; OCD 0.36; COD 0.18; R1L 1.09; R2L 1.31; R3L 0.32; A1L 3.01; A2L 1.59; A3L 1.52; A4L 1.56; PnL 2.45; PnW 2.74; APL 0.79; PPL 1.67; HeL 8.56; HeW 2.67; Sc 6.69; R+M 4.67; HWL 5.68; HWW 1.68; AFL 3.37; ATL 4.00; MFL 2.77; MTL 3.47; PFL 3.94; PTL 5.44. ***Paratype*** (♂): BL 9.77; HL 1.91; AoL 0.64; AoW 0.54; PoL 0.78; PoW 0.79; OE 1.05; IE 0.53; ED 0.63; OD 0.14; OCD 0.38; COD 0.19; R1L 1.05; R2L 1.31; R3L 0.29; A1L 3.48; A2L 1.62; A3L 1.49; A4L n/a; PnL 2.12; PnW 2.57; APL 0.59; PPL 1.52; HeL 8.45; HeW 2.79; Sc 6.78; R+M 4.52; AFL 3.44; ATL 4.06; MFL 2.90; MTL 3.52; PFL 3.99; PTL 5.36. ***Paratypes*** (♀). BL 10.61–10.75; HL 1.94–1.95; AoL 0.67; AoW 0.54–0.57; PoL 0.70–0.76; PoW 0.79–0.82; OE 1.05; IE 0.54–0.55; ED 0.58–0.62; OD 0.16; OCD 0.36–0.38; R1L 1.05–1.06; R2L 1.27–1.31; R3L 0.31–0.34; A1L 2.92–2.97; A2L 1.57–1.61; A3L 1.36–1.43; A4L 1.60–1.64; PnL 2.36–2.38; PnW 2.58–2.74; APL 0.72–0.84; PPL 1.52–1.66; HeL 8.65–8.88; HeW 2.75–2.98; Sc 6.30–6.49; R+M 4.40–4.48; HWL 5.78–5.89; HWW 1.79–1.91; AFL 3.31–3.33; ATL 3.78–3.93; MFL 2.70–2.78; MTL 3.42–3.51; PFL 3.69–3.87; PTL 4.91–5.35.

####### Distribution.

Vietnam, Central Highlands (Gia Lai, Dak Lak).

####### Type locality.

Vietnam, Central Highlands, Dak Lak Province, Chu Yang Sin National Park.

####### Etymology.

This new species is named after griseous pubescence on the pronotum.

###### ﻿ Biasticus luteicollis

Taxon classificationAnimaliaHemipteraReduviidae

Ha, Truong & Ishikawa sp. nov.

4C845DA7-AD00-5C4E-B3C1-6F7624A3CC19

https://zoobank.org/752E02B7-586A-4A9C-8D70-09DE6199F2EE

Figs 5C , 6J–N , 19 , 20 , 21

####### Type material.

***Holotype*.** ♂; HNL2018-025; Vietnam, Dak Lak Province, Chu Yang Sin National Park; 09.v.2018; X. L. Truong leg.; NSMT. ***Paratypes*.** 1♀; TXL2016-616; Vietnam, Dak Lak Province, Chu Yang Sin National Park; 05.v.2016; X. L. Truong leg.; IEBR. 1♂; TXL2016-617; Vietnam, Dak Lak Province, Chu Yang Sin National Park; 05.v.2016; X. L. Truong leg.; IEBR. 4♀; HNL2018-078; HNL2018-080; HNL2018-081; HNL2018-082; Vietnam, Gia Lai Province, Kon Chu Rang Nature Reserve; 08.v.2018; X. L. Truong leg.; IEBR. 4♂; HNL2018-079; HNL2018-083; HNL2018-084; HNL2018-085; Vietnam, Gia Lai Province, Kon Chu Rang Nature Reserve; 08.v.2018; X. L. Truong leg.; IEBR. 1♂; HNL2018-086; Vietnam, Gia Lai Province, Kon Chu Rang Nature Reserve; 08.v.2018; X. L. Truong leg.; NSMT. 1♀; HNL2018-017; Vietnam, Dak Lak Province, Chu Yang Sin National Park; 09.v.2018; X. L. Truong leg.; VNMN. 3♀; HNL2018-018; HNL2018-019; HNL2018-020; Vietnam, Dak Lak Province, Chu Yang Sin National Park; 09.v.2018; X. L. Truong leg.; IEBR. 3♀; HNL2018-021; HNL2018-023; HNL2018-024; Vietnam, Dak Lak Province, Chu Yang Sin National Park; 09.v.2018; X. L. Truong leg.; NSMT. 1♂; HNL2018-022; Vietnam, Dak Lak Province, Chu Yang Sin National Park; 09.v.2018; X. L. Truong leg.; NSMT.

####### Diagnosis.

Body shiny luteous; first visible labial segment and base of second visible labial segment luteous; apical 2/3 of second visible labial segment to third visible labial segment yellowish brown or brown; posterior pronotal lobe luteous; scutellum dark brown in basal half and luteous in apical half, with median pale brownish luteous portion; femora luteous with dark brown or yellowish brown suffusions at apex and sometimes at middle.

This species is most similar to *Biasticusflavus* (Distant, 1903) in general appearance, especially in coloration and color pattern of the pronotum, scutellum, and abdomen. However, the new species can be easily separated from the latter by a combination of the following characters: femora luteous with some dark brown or yellowish brown suffusions at apex and sometimes at middle (in *B.flavus* uniformly blackish brown) and abdominal sternites luteous without blackish brown or black suffusion (in *B.flavus* abdominal sternites luteous with blackish brown or black segmental transverse stripes laterally).

####### Description.

**Male description. *Coloration*.** Body shiny luteous. Head dorsum, except antenniferous tubercle and neck, black; clypeus, antenniferous tubercle, and labrum brown; maxillary plate, gena, and head venter (except anterior margin of maxillary plate and anterior margin of gena) pale yellowish brown; anterior margin of maxillary plate and anterior margin of gena yellowish brown; first visible labial segment and base of second visible labial segment luteous; apical two-thirds of second visible labial segment to third visible labial segment yellowish brown or brown; a luteous suffusion present above upper margin of compound eye; a luteous stripe running along dorsal margin of compound eye and between postero-upper corner of compound eye and ocellus; area around lateral ocellus with yellowish brown suffusion; a longitudinally elongated luteous suffusion present between lateral ocelli; neck yellowish brown. Scape, except base, yellowish brown; base of scape blackish brown; pedicel, first and second flagellomeres dark brown. Collar, anterolateral angle, and posterior pronotal lobe pale luteous; anterior pronotal lobe, except lateral margin, shiny dark brown, sometimes with luteous suffusion centrally; lateral margin of anterior pronotal lobe, acetabulum, thoracic sterna, stridulatory sulcus, coxae, and trochanters pale luteous; coxae and trochanters sometimes with dark brown or yellowish brown spots; pleura, except lower half of propleuron, dark brown; lower half of propleuron pale luteous; scutellum dark brown in basal half and luteous in apical half, with median pale brownish luteous portion; femora luteous; fore femora apically dark brown, sometimes with dark brown or yellowish brown suffusion; mid and hind femora apically and medially yellowish brown; tibiae dark brown. Corium and clavus yellow or brownish yellow; membrane bronzy brown, semi-hyaline. Hind wings faintly semi-hyaline. Abdominal mediotergites, except posterior half of mediotergite VII, brownish yellow; posterior half of mediotergite VII luteous; abdominal sternites pale luteous; connexivum pale luteous, with segmentally dark brown suffusions. Pygophore ventrally luteous; paramere semi-hyaline, luteous.

***Structure*.** Body medium-sized (BL = 9.19–10.21 mm), elongate, and somewhat robust. Head subelongate and robust (HL/PoW = 2.35–2.48), as long as or a little shorter than pronotum (HL/PnL = 0.84–1.03); postocular area of head sub-globose (PoL/PoW = 0.77–0.93), distinctly wider than anteocular area (PoW/AoW = 1.36–1.43), nearly as long as anteocular area (PoL/AoL = 0.84–1.03), constricted behind compound eyes, with a wide and deep interocular sulcus; neck short. Compound eyes protruding laterally, nearly globose, with posterior margin sub-straight and sometimes concave; lateral ocelli produced, elevated behind interocular sulcus, widely separated from each other (OCD/PoW = 0.42–0.48); interspace between lateral ocelli wider than distance between compound eye and lateral ocellus (OCD/COD = 1.95–2.36). First visible labial segment shorter than second segment (R1L/R2L = 0.79–0.82), longer than anteocular area of head (R1L/AoL = 1.39–1.51), not extending beyond level of middle of compound eye when labium laid backward; proportional average length of first to third visible labial segments 1.1:1.3:0.4. Scape ~ 1.4 × as long as head; pedicel slightly shorter than first flagellomere and nearly 0.7 × as long as second flagellomere; proportional average length of scape, pedicel, first and second flagellomeres 2.9:1.4:1.5:2.0. Collar very thick in dorsal view, with anterolateral angle roundly produced laterad; anterior pronotal lobe round and bulged, slightly rough, with middle longitudinal sulcus deep and narrow posteriorly; posterior pronotal lobe shallowly depressed on disc, abundantly punctured, with slightly swollen anteromedial elevation (never sulcate or concave); humerus roughly triangular, with round apex; posterior margin of pronotum concave; posterior angles round, slightly exceeded to posterior margin of pronotum. Scutellum triangular, somewhat triangularly depressed basally, apically produced and sloping downward; posterior apex round; anterior margin slightly convex. Femora thick, apically moderately nodulose; fore femora very slightly incrassated, thicker than mid and hind femora. Hemelytra surpassing beyond apex of abdomen when fully closed, 0.8 × as long as body length; discal cell nearly parallelogram-shaped, twice as long as width; Sc 0.8 × as long as hemelytron length, 1.5 × as long as R + M. Hind wing ~ 3.3 × as long as maximum width. Connexivum slightly dilated and ascending with segmental incisures; pygophore ovoid; median process of pygophore (mpp) broad and low, 0.2 × as long as wide, with apical margin weakly and continuously convex, and with apicolateral corner slightly produced laterad and pointed; paramere long, slender, clavate, somewhat curved medially, and apically subnodulose and round (Figs 6J, 21B–E). Aedeagus in dorsal view ovoid, dorsally sclerotized (Figs 6K, 21F) and in lateral view long and narrow (Figs 6L, 21G); articulatory apparatus (aa) in ventral view with relatively broad basal plates jointly forming a V-shape, and in lateral view arched moderately (Figs 6L, N, 21G, I); dorsal phallothecal sclerite (dps) in lateral view with posteromedian part strongly produced posterodorsad (Figs 6L, 21G); spoon-like sclerites (sps) semi-hyaline and covered with tiny blunt prickles; both membranous sac-like lobes laterally produced; distal dorsal lobe of endosoma (ddl) round, with membranous surface covered with small but distinct prickles (Figs 6M, 21J).

***Vestiture*.** Body clothed with cream-yellow setation. Head dorsum (except antenniferous tubercle, posterior base of clypeus, and gena) covered with short thick erect setae; area around antenniferous tubercle, posterior margin of clypeus, head venter, and lateral area of postocular area of head covered with short bent pubescence; head venter somewhat with a few long bent setae; maxillary plate without setae; first visible labial segment basally covered with a few erect setae; second and third visible labial segments without setae; scape with a few tiny erect setae; pedicel, first and second flagellomeres densely covered with short slender oblique setae. Anterior margin of collar densely covered with very short erect setae; dorsum of collar with bent cream-yellow pubescence; anterolateral angle and lateral region of collar with long thick erect setae, one of them placed at tip of anterolateral angle; pronotum and scutellum with long thick erect setae; lateral region of slopes of scutellum covered with long slender bent setae; posterior apex of scutellum with a pinch of long slender bent setae and long thick vertical setae; prosternum, except posterior margin, covered with long thick erect setae; posterior margin of prosternum, meso- and metasterna, pleura, and coxae abundantly covered with short bent pubescence. Corium densely covered with bent setae along anterior margin, centrally covered with short slender oblique setae. Abdomen sometimes with erect setae, slightly denser in posterior margin of abdomen; anterior margin of mediotergite I+II, anterior margin of sternite II, and lateral region of laterotergite II with very long slender erect setae; pygophore posteroventrally with some long erect setae and some bent slender setae; paramere with long thick erect setae.

**Female description.** General external morphology similar to that of the male.

***Coloration*.** Almost similar to male but slightly darker than male. Abdominal mediotergites, except posterior half of mediotergite VII, dark brown and darker backward; posterior half of mediotergite VII luteous; external genitalia pale luteous.

***Structure*.** Almost similar to male but slightly larger than male. Body medium-sized (BL = 10.11–10.91 mm). Proportional average length of first to third visible labial segments 1.1:1.4:0.4. Scape ~ 1.3 × as long as head; pedicel ~ as long as first flagellomere and ~ 0.8 × as long as second flagellomere; proportional average length of scape, pedicel, first and second flagellomeres 2.7:1.3:1.3:1.7. Sc 0.8 × as long as hemelytron length, and 1.5 × as long as R + M. Hind wing ~ 3.1 × as long as maximum width. Abdominal laterotergite VIII (AL8) with very thick posterior margin (0.09 mm); abdominal sternite VII (AS7) forming a wide subrectangular concavity, with posteromedian margin almost straight, with inner posterolateral margin weakly sinuous; gonocoxa VIII (Gc8) broad, ~ 1.2 × wider than length, slightly opened inward for gonapophyses VIII (Gp8), sinuate along outer lateral margin, almost horizontal or weakly sinuate along posterior margin, weakly produced posteromesad and forming a blunt apex at apical inner corner, and with inner margin strongly incurved in its posterior 2/3; Gp8 small and subtriangular, ~ 3 × longer than width; gonoplac (Gpl) V-shaped, with maximum thick ~ 0.05 mm.

***Vestiture*.** Almost similar to male. Abdominal laterotergite VIII (AL8) and tergite IX (AT9) with some long thick erect setae and with shorter setae; apex of abdomen with some very long thick erect setae.

####### Measurements.

All dimensions are given in mm. ***Holotype*** (♂): BL 9.29; HL 2.01; AoL 0.75; AoW 0.60; PoL 0.62; PoW 0.81; OE 1.12; IE 0.58; ED 0.63; OD 0.16; OCD 0.35; COD 0.18; R1L 1.03; R2L 1.30; R3L 0.32; A1L 2.85; A2L 1.41; A3L 1.55; A4L 2.07; PnL 2.21; PnW 2.64; APL 0.90; PPL 1.31; HeL 7.26; HeW 2.47; Sc 5.66; R+M 3.84; HWL 4.92; HWW 1.54; AFL 3.33; ATL 3.87; MFL 2.46; MTL 3.24; PFL 3.58; PTL 4.84. ***Paratypes*** (♂): BL 9.19–10.45; HL 1.94–2.12; AoL 0.70–0.79; AoW 0.57–0.62; PoL 0.64–0.77; PoW 0.80–0.86; OE 1.08–1.17; IE 0.55–0.59; ED 0.60–0.64; OD 0.14–0.19; OCD 0.34–0.41; COD 0.15–0.18; R1L 1.03–1.14; R2L 1.26–1.41; R3L 0.32–0.46; A1L 2.68–3.06; A2L 1.30–1.55; A3L 1.37–1.61; A4L 1.74–2.17; PnL 1.95–3.10; PnW 2.42–2.74; APL 0.74–0.99; PPL 1.22–2.25; HeL 7.26–7.88; HeW 2.23–2.63; Sc 5.32–6.09; R+M 3.44–4.08; HWL 4.92–5.22; HWW 1.46–1.62; AFL 3.15–3.46; ATL 3.78–4.08; MFL 2.33–2.69; MTL 2.73–3.50; PFL 3.56–3.91; PTL 4.80–5.10. ***Paratypes*** (♀): BL 10.11–10.91; HL 2.00–2.10; AoL 0.73–0.79; AoW 0.60–0.63; PoL 0.67–0.76; PoW 0.82–0.87; OE 1.06–1.15; IE 0.55–0.60; ED 0.59–0.63; OD 0.15–0.19; OCD 0.36–0.46; COD 0.18–0.20; R1L 1.08–1.15; R2L 1.31–1.41; R3L 0.32–0.37; A1L 2.52–2.83; A2L 1.26–1.35; A3L 1.23–1.41; A4L 1.61–1.87; PnL 2.09–2.47; PnW 2.24–3.65; APL 0.70–0.88; PPL 1.39–1.65; HeL 7.66–8.21; HeW 2.60–2.92; Sc 5.86–6.32; R+M 4.01–4.45; HWL 5.25–6.82; HWW 1.67–2.15; AFL 3.00–3.33; ATL 3.63–4.07; MFL 2.31–3.13; MTL 2.94–3.83; PFL 3.33–3.80; PTL 4.61–5.13.

####### Distribution.

Vietnam, Central Highlands (Dak Lak, Gia Lai).

####### Type locality.

Vietnam, Central Highlands, Dak Lak Province, Chu Yang Sin National Park.

####### Etymology.

The new species is named after its yellow posterior pronotal lobe.

**Figure 10.**
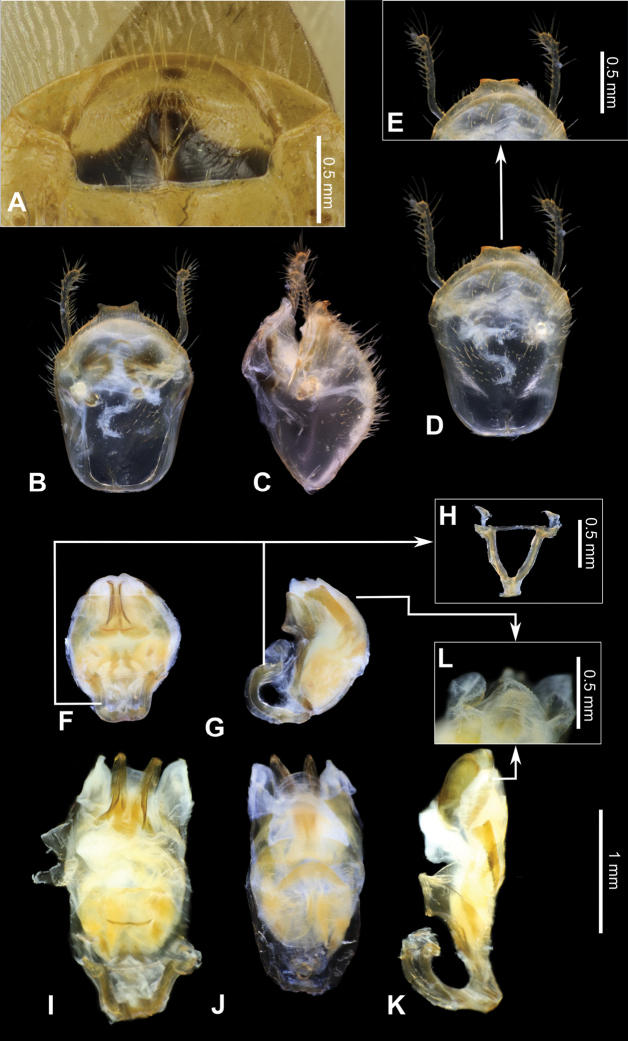
*Biasticusflavus* (Distant, 1903) **A** female genitalia in ventral view, ♀, LA-Redu-2004-006 **B–L** male genitalia, paratype, ♂, HEM-TH2004-018 **B–E** pygophore with parameres **B** dorsal view **C** lateral view **D** ventral view **E** apical portion of pygophore showing median process (mpp) and parameres **F–L** phallus **F** dorsal view **G** lateral view **H** articulatory apparatus (aa) **I** phallus with endosoma semi-everted, dorsal view **J** aedeagus with endosoma semi-everted, ventral view **K** phallus with endosoma semi-everted, lateral view **L** distal dorsal lobe of endosoma (ddl).

**Table 3.** Correlation and contribution in the percentage for the first and second dimensions (PC1 and PC2) of Principle Component Analysis (PCA) of the 10 most contributing morphometric characters based on female and male datasets (upper and lower tables, respectively).

**Female dataset**										
	A2L	HeL	Sc	A4L	mGr	mBr	HL	AFL	AoL	A1L
Dim.1 (PC1)	0.855	0.947	0.872	-0.783	-0.732	0.842	-0.235	0.102	-0.109	0.413
Dim.2 (PC2)	-0.139	0.189	0.412	-0.428	0.182	-0.008	0.689	-0.512	0.833	-0.542
Contribution (%)	4.720	4.701	4.650	4.614	4.612	4.531	4.494	4.428	4.326	4.229
**Male dataset**										
	PTL	PFL	mGr	MFL	A1L	AFL	ATL	OD	R2L	OE
Dim.1 (PC1)	0.940	0.891	-0.060	0.921	0.926	0.824	0.747	-0.737	0.685	-0.318
Dim.2 (PC2)	0.243	0.166	0.880	0.190	-0.117	0.390	0.535	-0.148	0.600	0.727
Contribution (%)	4.130	4.109	4.083	4.061	4.051	3.983	3.942	3.940	3.913	3.895

**Figure 11.**
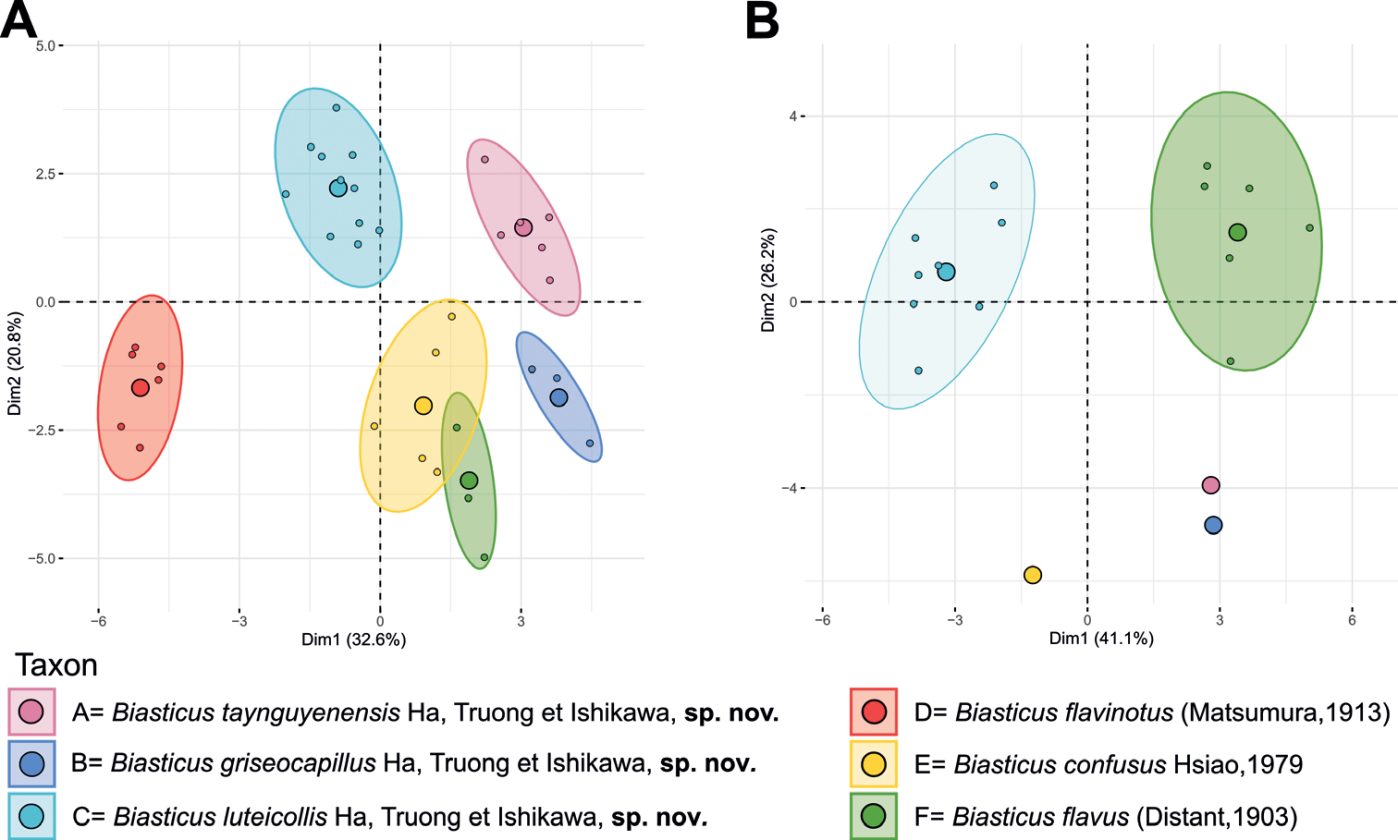
2D PCA-plots from morphological dataset of the female adults (**A**) and male adults (**B**) of *Biasticus* collected from Vietnam and its surrounding areas.

**Figure 12.**
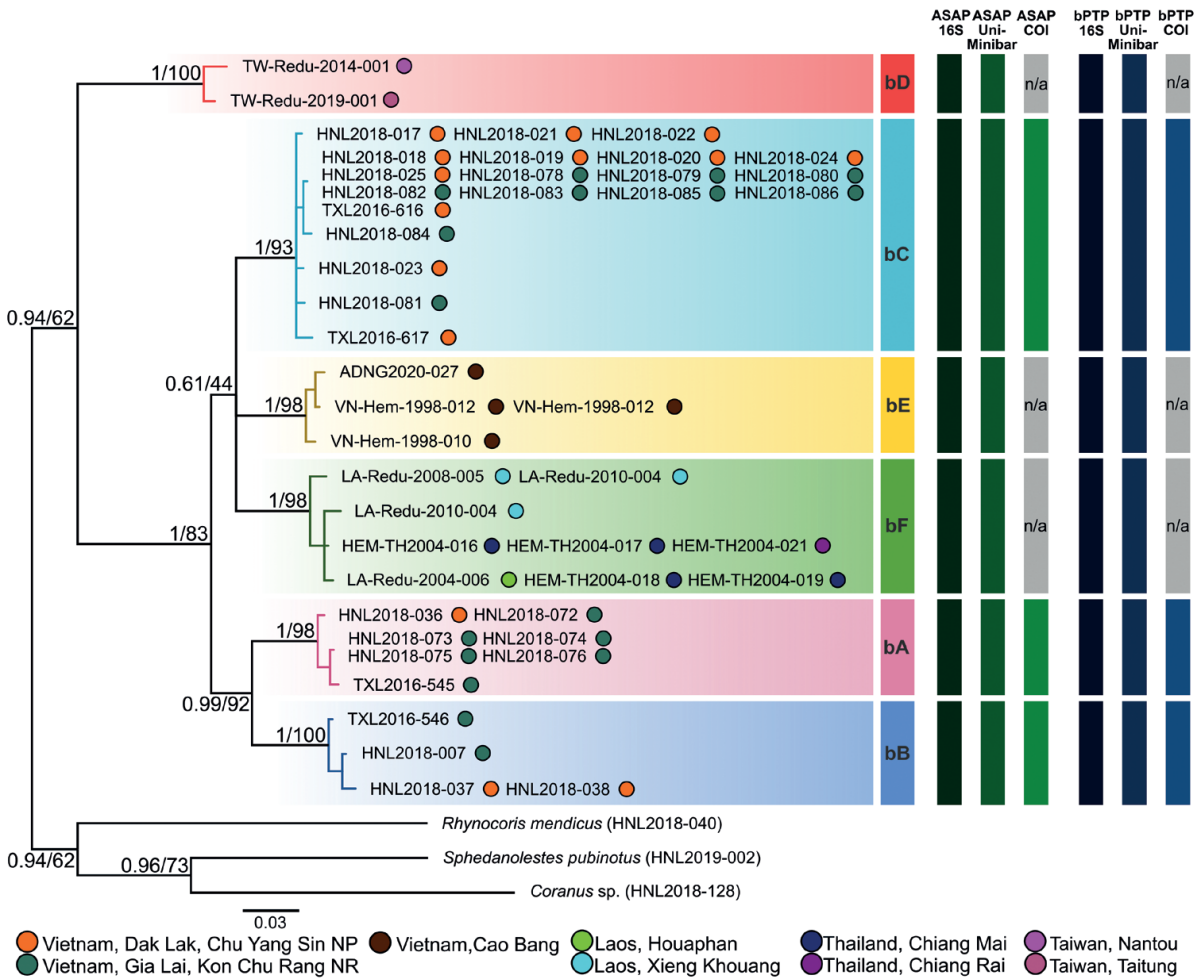
Bayesian inference phylogenetic tree based on the concatenated 16S + Uni dataset (661 bp) of *Biasticus* species collected from Vietnam and its surrounding areas. Posterior probability values and bootstrap values (in %) were given beside the basal nodes. The tips are labeled with the specimen IDs. The circles after specimen IDs showed the collecting localities.

**Figure 13.**
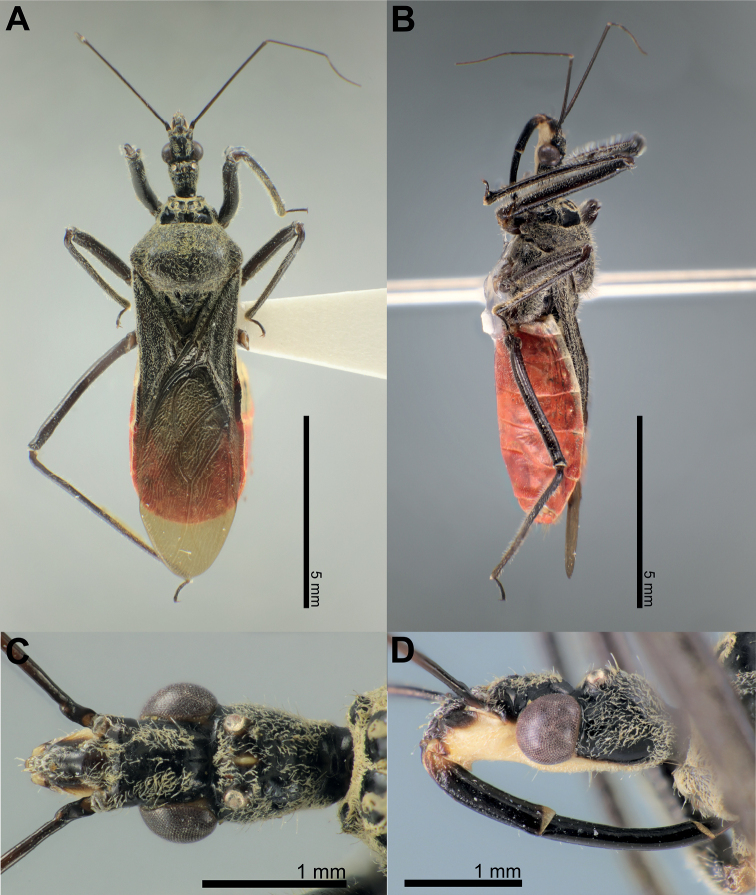
*Biasticustaynguyenensis* Ha, Truong & Ishikawa, sp. nov., holotype, ♀, HNL2018-073 **A** body in dorsal view **B** body in lateral view **C** head in dorsal view **D** head in lateral view.

**Figure 14.**
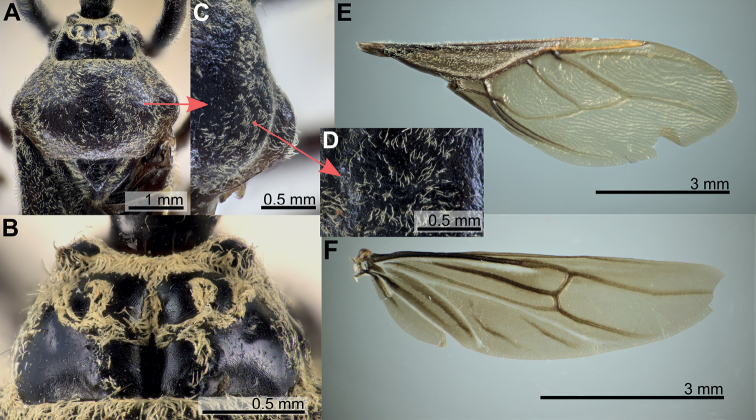
*Biasticustaynguyenensis* Ha, Truong & Ishikawa, sp. nov., holotype, ♀, HNL2018-073 **A** pronotum in dorsal view **B** anterior pronotal lobe in dorsal view **C, D** setae on posterior pronotum **E** right hemelytron **F** right hind wing.

**Figure 15.**
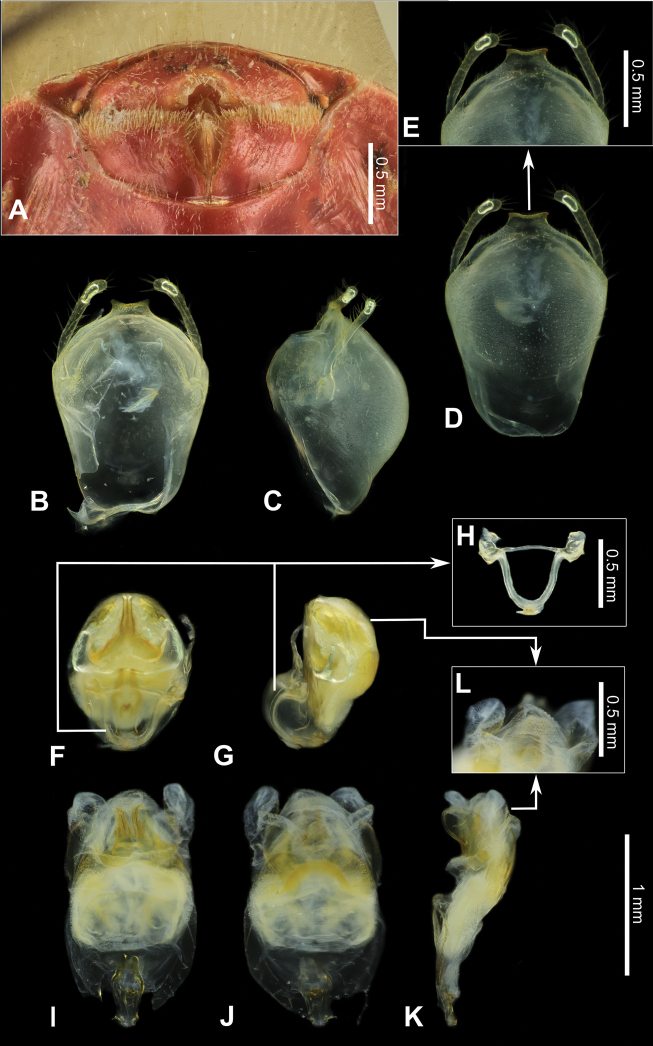
*Biasticustaynguyenensis* Ha, Truong & Ishikawa, sp. nov. **A** female genitalia in ventral view, holotype, ♀, HNL2018-073 **B–L** male genitalia, paratype, ♂, TXL2016-545 **B–E** pygophore with parameres **B** dorsal view **C** lateral view **D** ventral view **E** apical portion of pygophore, showing median process (mpp) and parameres **F–L** phallus **F** dorsal view **G** lateral view **H** articulatory apparatus (aa) **I** aedeagus with endosoma semi-everted dorsal view **J** aedeagus with endosoma semi-everted, ventral view **K** aedeagus with endosoma semi-everted, lateral view **L** distal dorsal lobe of endosoma (ddl).

**Figure 16.**
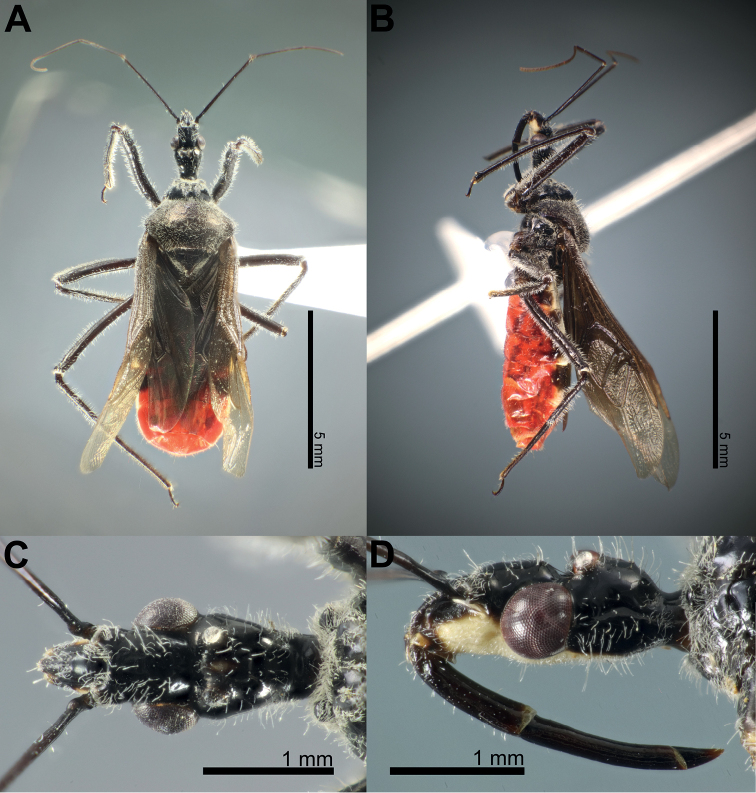
*Biasticusgriseocapillus* Ha, Truong & Ishikawa, sp. nov., holotype, ♀, HNL2018-038 **A** body in dorsal view **B** body in lateral view **C** head in dorsal view **D** head in lateral view.

**Figure 17.**
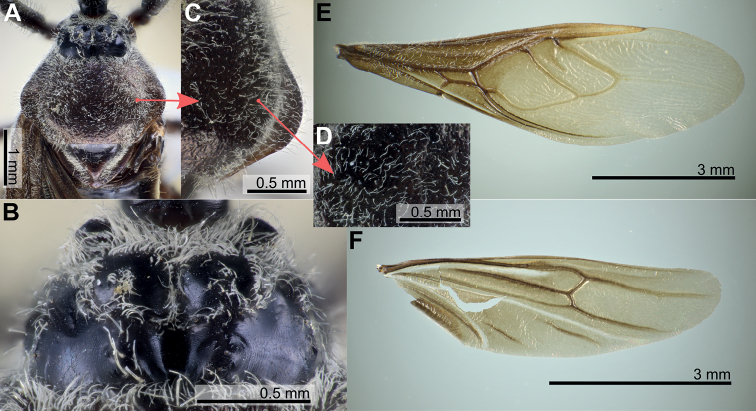
*Biasticusgriseocapillus* Ha, Truong & Ishikawa, sp. nov., holotype, ♀, HNL2018-038 **A** pronotum in dorsal view **B** anterior pronotal lobe in dorsal view **C, D** setae on posterior pronotum **E** right hemelytron **F** right hind wing.

**Figure 18.**
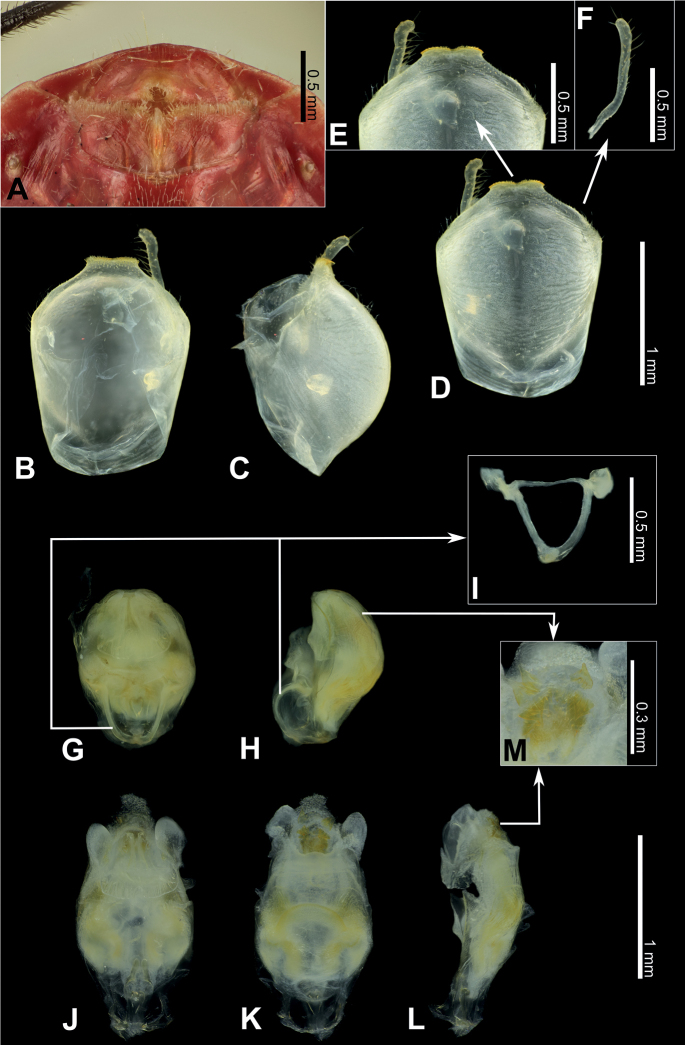
*Biasticusgriseocapillus* Ha, Truong & Ishikawa, sp. nov. **A** female genitalia in ventral view, holotype, ♀, HNL2018-038 **B–M** male genitalia, paratype, ♂, TXL2016-546 **B–F** pygophore with paramere **B** dorsal view **C** lateral view **D** ventral view **E** apical portion of pygophore, showing median process (mpp) and paramere **F** left paramere **G–M** phallus **G** dorsal view **H** lateral view **I** articulatory apparatus (aa) **J** aedeagus with endosoma semi-everted, dorsal view **K** aedeagus with endosoma semi-everted, ventral view **L** aedeagus with endosoma semi-everted, lateral view **M** distal dorsal lobe of endosoma (ddl).

**Figure 19.**
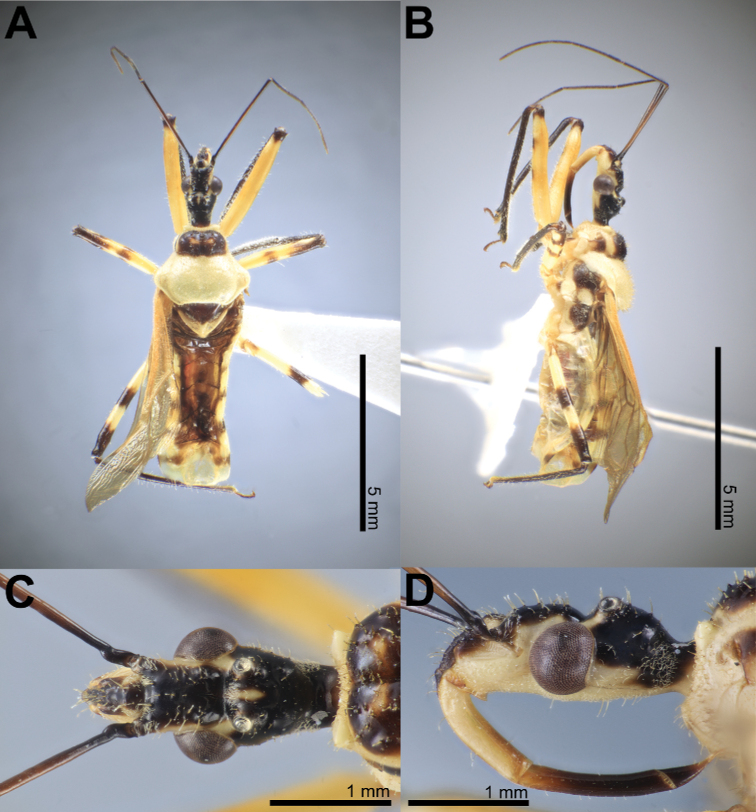
*Biasticusluteicollis* Ha, Truong & Ishikawa, sp. nov., holotype, ♂, HNL2018-025 **A** body in dorsal view **B** body in lateral view **C** head in dorsal view **D** head in lateral view.

**Figure 20.**
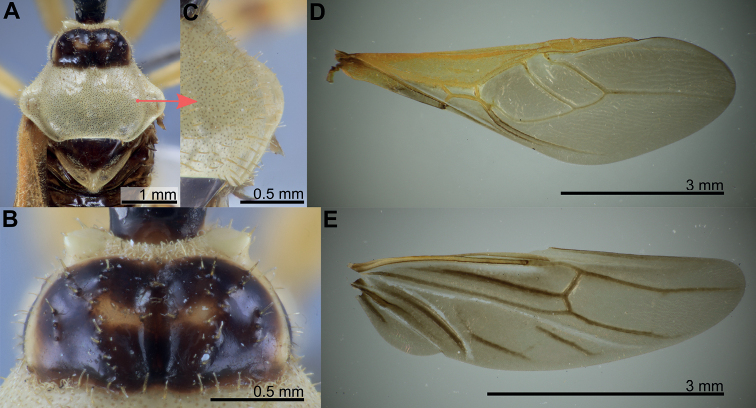
*Biasticusluteicollis* Ha, Truong & Ishikawa, sp. nov., holotype, ♂, HNL2018-025 **A** pronotum in dorsal view **B** anterior pronotal lobe in dorsal view **C** setae on posterior pronotum **D** right hemelytron **E** right hind wing.

**Figure 21.**
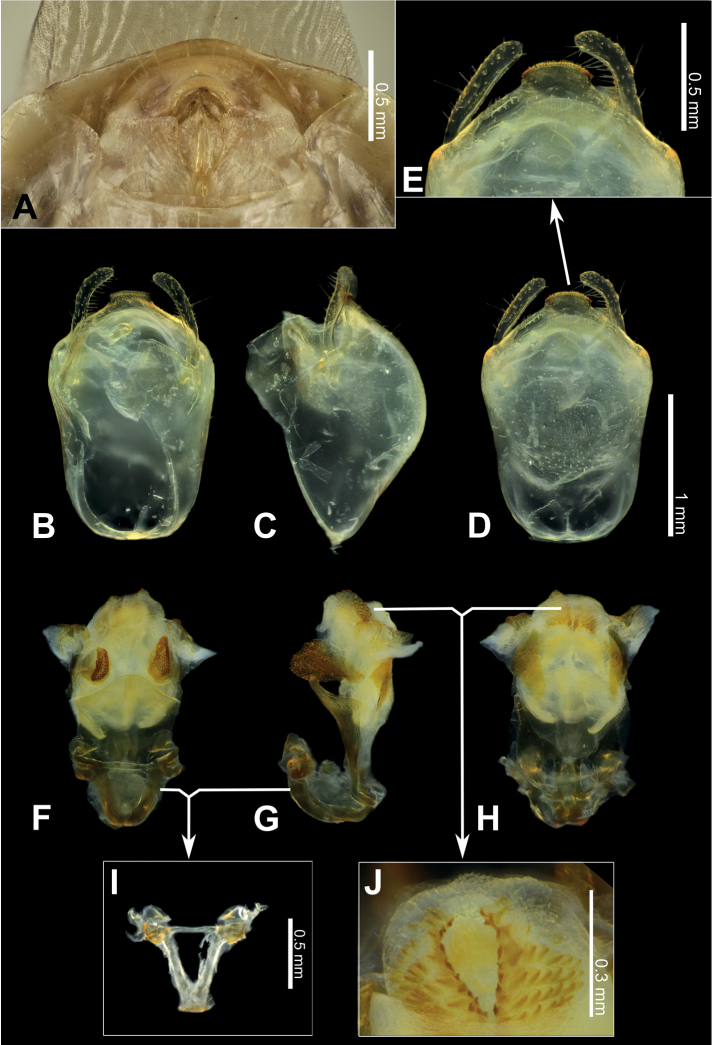
*Biasticusluteicollis* Ha, Truong & Ishikawa, sp. nov. **A** female external genitalia in ventral view, paratype, ♀, HNL2018-024 **B–J** male genitalia, holoype, ♂, HNL2018-025 **B–E** pygophore with parameres **B** dorsal view **C** lateral view **D** ventral view **E** apical portion of pygophore, showing median process (mpp) and parameres **F–H** phallus **F** phallus with endosoma semi-everted, dorsal view **G** phallus with endosoma semi-everted, lateral view **H** phallus with endosoma semi-everted, ventral view **I** articulatory apparatus (aa) **J** distal dorsal lobe of endosoma (ddl).

### ﻿Key to the species of the genus *Biasticus* Stål, 1867 from Vietnam

**Table:** 

1	Posterior pronotal lobe pale luteous to yellow	**2**
–	Posterior pronotal lobe brown to black	**4**
2	Connexivum sanguineous; scutellum blackish brown or black with posterior apex blackish brown or black	***Biasticusflavinotus* (Matsumura, 1913)**
–	Connexivum pale luteous to luteous; scutellum dark brown with posterior apex luteous	**3**
3	Abdominal sternites luteous with some blackish brown or black segmental transverse stripes laterally; femora uniformly blackish brown or brown	***Biasticusflavus* (Distant, 1903)**
–	Abdominal sternites pale luteous without blackish brown or black segmental transverse stripes laterally; femora luteous with some dark brown or yellowish brown suffusions	***Biasticusluteicollis* Ha, Truong & Ishikawa, sp. nov.**
4	Abdominal sternites luteous to sanguineous; anterior pronotal lobe without row of bent setae; pedicel as long as first flagellomere and shorter than second flagellomeres	***Biasticusconfusus* Hsiao et al., 1979**
–	Abdominal sternites sanguineous; anterior pronotal lobe with some rows of bent setae; pedicel slightly longer than first flagellomere and as long as second flagellomere	**5**
5	Posterior pronotal lobe densely covered with short bent cream-yellow setae, interspersed with long erect setae; apical margin of median process of pygophore weakly concave as a whole	***Biasticustaynguyenensis* Ha, Truong & Ishikawa, sp. nov.**
–	Posterior pronotal lobe densely covered with short bent griseous setae, sometimes interspersed with long griseous setae; apical margin of median process of pygophore weakly convex as a whole and slightly emarginate at middle	***Biasticusgriseocapillus* Ha, Truong & Ishikawa, sp. nov.**

## Supplementary Material

XML Treatment for Biasticus taynguyenensis

XML Treatment for Biasticus griseocapillus

XML Treatment for Biasticus luteicollis

## Supplementary materials

Supplementary material 1 R-script used for morphometric analyses https://doi.org/10.3897/zookeys.1118.83156.suppl1 Data type statistical data https://binary.pensoft.net/file/734765This dataset is made available under the Open Database License (http://opendatacommons.org/licenses/odbl/1.0/). The Open Database License (ODbL) is a license agreement intended to allow users to freely share, modify, and use this Dataset while maintaining this same freedom for others, provided that the original source and author(s) are credited.Author: Ngoc Linh Ha

Supplementary material 2 Female morphometric dataset https://doi.org/10.3897/zookeys.1118.83156.suppl2 Data type species data https://binary.pensoft.net/file/734766This dataset is made available under the Open Database License (http://opendatacommons.org/licenses/odbl/1.0/). The Open Database License (ODbL) is a license agreement intended to allow users to freely share, modify, and use this Dataset while maintaining this same freedom for others, provided that the original source and author(s) are credited.Author: Ngoc Linh Ha

Supplementary material 3 Male morphometric dataset https://doi.org/10.3897/zookeys.1118.83156.suppl3 Data type species data https://binary.pensoft.net/file/734767This dataset is made available under the Open Database License (http://opendatacommons.org/licenses/odbl/1.0/). The Open Database License (ODbL) is a license agreement intended to allow users to freely share, modify, and use this Dataset while maintaining this same freedom for others, provided that the original source and author(s) are credited.Author: Ngoc Linh Ha
