# ﻿Corrigendum: ﻿Three new species of the genus *Biasticus* Stål, 1867 (Insecta, Heteroptera, Reduviidae, Harpactorinae) from Central Highlands, Vietnam. ZooKeys 1118: 133–180. https://doi.org/10.3897/zookeys.1118.83156

**DOI:** 10.3897/zookeys.1134.97678

**Published:** 2022-12-09

**Authors:** Ngoc Linh Ha, Xuan Lam Truong, Tadashi Ishikawa, Weeyawat Jaitrong, Chi Feng Lee, Bounsanong Chouangthavy, Katsuyuki Eguchi

**Affiliations:** 1 Department of Biological Sciences, Graduate School of Science, Tokyo Metropolitan University, Minami-osawa 1-1, Hachioji, Tokyo 192-0397, Japan Tokyo Metropolitan University Tokyo Japan; 2 Institute of Ecology and Biological Resources, Vietnam Academy of Science and Technology, 18 Hoang Quoc Viet Road, Nghia Do, Cau Giay, Hanoi, Vietnam Institute of Ecology and Biological Resources, Vietnam Academy of Science and Technology Hanoi Vietnam; 3 Graduate University of Science and Technology, Vietnam Academy of Science and Technology, 18 Hoang Quoc Viet Road, Nghia Do, Cau Giay, Hanoi, Vietnam Graduate University of Science and Technology Hanoi Vietnam; 4 Faculty of Agriculture, Tokyo University of Agriculture, Atsugi, Kanagawa, Japan Tokyo University of Agriculture Atsugi Japan; 5 Office of Natural Science Research, National Science Museum, 39 Moo 3, Khlong 5, Khlong Luang, Pathum Thani, 12120 Thailand National Science Museum Pathum Thani Thailand; 6 Applied Zoology Division, Taiwan Agricultural Research Institute, Taichung 413, Taiwan Taiwan Agricultural Research Institute Taichung Taiwan; 7 Plant Protection Unit, Department of Plant Science, Faculty of Agriculture, National University of Laos, P.O. Box 7322, Vientiane, Lao People’s Democratic Republic National University of Laos Vientiane Lao People's Democratic Republic

We recently published the description of three new species of the genus *Biasticus* Stål, 1867 (Insecta, Heteroptera, Reduviidae, Harpactorinae) from Central Highlands, Vietnam (Ha NL, Truong XL, Ishikawa T, Jaitrong W, Lee CF, Chouangthavy B, Eguchi K 2022). However, the first author made mistakes when indicating the deposition of the holotype and paratype of the three new species regardless of some regulations and agreements about those specimens. In this corrigendum, we made a revision of the status of the holotype and paratype deposition.

**Table 1.** The data of specimens used in this study. Abbreviations and symbols: n/a: no data; HU, Matsumura Collection at the Laboratory of Systematic Entomology, Department of Agriculture, Hokkaido University, Sapporo, Japan; IEBR, Institute of Ecology and Biological Resources, Vietnam Academy of Science and Technology, Vietnam; NSMT, National Museum of Nature and Science, Tokyo, Japan; NSM, Department of Entomology, Zoological Research Division, Office of Natural Science Research, National Science Museum, Thailand; NUOL-FA, Faculty of Agriculture, National University of Laos, Laos P.D.R; TARI-AZ, Applied Zoology Division, Taiwan Agricultural Research Institute Insect Collection, Taiwan Agricultural Research Institute, Taiwan; VNMN, Vietnam National Museum of Nature, Vietnam Academy of Science and Technology, Vietnam; *, tentatively held by HNL (first author); bA–bF, morphospecies code (see in the text).

Morphospecies	Specimen code	Collecting date	Locality	Sex	Accession numbers			Depository
					16S	Uni-Minibar (COI)	COI	
*Biasticus* (ingroups)								
*B.taynguyenensis* Ha, Truong & Ishikawa, sp. nov. [bA] [Paratype]	HNL2018-036	09.v.2018	Vietnam, Dak Lak	♀	OM908207	ON542864	OM868188	IEBR*
*B.taynguyenensis* Ha, Truong & Ishikawa, sp. nov. [bA]	HNL2018-072	08.v.2018	Vietnam, Gia Lai	♀	OM908210	ON542867	OM868178	NSMT
*B.taynguyenensis* Ha, Truong & Ishikawa, sp. nov. [bA] [Holotype]	HNL2018-073	08.v.2018	Vietnam, Gia Lai	♀	OM908211	ON542868	OM868192	IEBR*
*B.taynguyenensis* Ha, Truong & Ishikawa, sp. nov. [bA] [Paratype]	HNL2018-074	08.v.2018	Vietnam, Gia Lai	♀	OM908212	ON542869	OM868193	VNMN
*B.taynguyenensis* Ha, Truong & Ishikawa, sp. nov. [bA] [Paratype]	HNL2018-075	08.v.2018	Vietnam, Gia Lai	♀	OM908213	ON542870	OM868194	VNMN
*B.taynguyenensis* Ha, Truong & Ishikawa, sp. nov. [bA]	HNL2018-076	08.v.2018	Vietnam, Gia Lai	♀	ON554765	ON542871	n/a	NSMT
*B.taynguyenensis* Ha, Truong & Ishikawa, sp. nov. [bA] [Paratype]	TXL2016-545	28.iv.2016	Vietnam, Gia Lai	♂	OM908227	ON542894	OM868177	IEBR*
*B.griseocapillus* Ha, Truong & Ishikawa, sp. nov. [bB]	HNL2018-007	05.v.2018	Vietnam, Gia Lai	♀	OM908197	ON542854	OM868176	NSMT
*B.griseocapillus* Ha, Truong & Ishikawa, sp. nov. [bB] [Paratype]	HNL2018-037	09.v.2018	Vietnam, Dak Lak	♀	OM908208	ON542865	OM868189	IEBR*
*B.griseocapillus* Ha, Truong & Ishikawa, sp. nov. [bB] [Holotype]	HNL2018-038	09.v.2018	Vietnam, Dak Lak	♀	OM908209	ON542866	OM868191	IEBR*
*B.griseocapillus* Ha, Truong & Ishikawa, sp. nov. [bB] [Paratype]	TXL2016-546	28.iv.2016	Vietnam, Gia Lai	♂	OM908228	ON542895	OM868190	IEBR*
*B.luteicollis* Ha, Truong & Ishikawa, sp. nov. [bC] [Paratype]	HNL2018-017	09.v.2018	Vietnam, Dak Lak	♀	OM908198	ON542855	OM868179	VNMN
*B.luteicollis* Ha, Truong & Ishikawa, sp. nov. [bC]	HNL2018-018	09.v.2018	Vietnam, Dak Lak	♀	OM908199	ON542856	OM868180	IEBR*
*B.luteicollis* Ha, Truong & Ishikawa, sp. nov. [bC]	HNL2018-019	09.v.2018	Vietnam, Dak Lak	♀	OM908200	ON542857	OM868181	IEBR*
*B.luteicollis* Ha, Truong & Ishikawa, sp. nov. [bC] [Paratype]	HNL2018-020	09.v.2018	Vietnam, Dak Lak	♀	OM908201	ON542858	OM868182	IEBR*
*B.luteicollis* Ha, Truong & Ishikawa, sp. nov. [bC]	HNL2018-021	09.v.2018	Vietnam, Dak Lak	♀	OM908202	ON542859	OM868183	NSMT
*B.luteicollis* Ha, Truong & Ishikawa, sp. nov. [bC]	HNL2018-022	09.v.2018	Vietnam, Dak Lak	♂	OM908203	ON542860	OM868184	NSMT
*B.luteicollis* Ha, Truong & Ishikawa, sp. nov. [bC]	HNL2018-023	09.v.2018	Vietnam, Dak Lak	♀	OM908204	ON542861	OM868185	NSMT
*B.luteicollis* Ha, Truong & Ishikawa, sp. nov. [bC] [Paratype]	HNL2018-024	09.v.2018	Vietnam, Dak Lak	♀	OM908205	ON542862	OM868186	IEBR*
*B.luteicollis* Ha, Truong & Ishikawa, sp. nov. [bC] [Holotype]	HNL2018-025	09.v.2018	Vietnam, Dak Lak	♂	OM908206	ON542863	OM868187	IEBR*
*B.luteicollis* Ha, Truong & Ishikawa, sp. nov. [bC]	HNL2018-078	08.v.2018	Vietnam, Gia Lai	♀	OM908214	ON542872	OM868195	NSMT
*B.luteicollis* Ha, Truong & Ishikawa, sp. nov. [bC]	HNL2018-079	08.v.2018	Vietnam, Gia Lai	♂	OM908215	ON542873	OM868196	NSMT
*B.luteicollis* Ha, Truong & Ishikawa, sp. nov. [bC]	HNL2018-080	08.v.2018	Vietnam, Gia Lai	♀	OM908216	ON542874	OM868197	IEBR*
*B.luteicollis* Ha, Truong & Ishikawa, sp. nov. [bC]	HNL2018-081	08.v.2018	Vietnam, Gia Lai	♀	OM908217	ON542875	OM868198	IEBR*
*B.luteicollis* Ha, Truong & Ishikawa, sp. nov. [bC] [Paratype]	HNL2018-082	08.v.2018	Vietnam, Gia Lai	♀	OM908218	ON542876	OM868199	VNMN
*B.luteicollis* Ha, Truong & Ishikawa, sp. nov. [bC] [Paratype]	HNL2018-083	08.v.2018	Vietnam, Gia Lai	♂	OM908219	ON542877	OM868200	VNMN
*B.luteicollis* Ha, Truong & Ishikawa, sp. nov. [bC]	HNL2018-084	08.v.2018	Vietnam, Gia Lai	♂	OM908220	ON542878	OM868201	NSMT
*B.luteicollis* Ha, Truong & Ishikawa, sp. nov. [bC] [Paratype]	HNL2018-085	08.v.2018	Vietnam, Gia Lai	♂	OM908221	ON542879	OM868202	IEBR*
*B.luteicollis* Ha, Truong & Ishikawa, sp. nov. [bC] [Paratype]	HNL2018-086	08.v.2018	Vietnam, Gia Lai	♂	OM908222	ON542880	OM868203	IEBR*
*B.luteicollis* Ha, Truong & Ishikawa, sp. nov. [bC]	TXL2016-616	05.v.2018	Vietnam, Dak Lak	♀	OM908229	ON542896	OM868208	NSMT
*B.luteicollis* Ha, Truong & Ishikawa, sp. nov. [bC]	TXL2016-617	05.v.2018	Vietnam, Dak Lak	♂	OM908230	ON542897	OM868209	NSMT

***Biasticustaynguyenensis* Ha, Truong & Ishikawa, sp. nov.**

https://zoobank.org/290765E6-63AE-49F4-B835-B22A034E4DE7

Figs 5A, 6A–D, 13, 14, 15

**Type material. *Holotype*.** ♀; HNL2018-073; Vietnam, Gia Lai Province, Kon Chu Rang Nature Reserve; 08.v.2018; X. L. Truong leg.; **IEBR. *Paratypes*.** 1♂; TXL2016-545; Vietnam, Gia Lai Province, Kon Chu Rang Nature Reserve; 28.iv.2016; X. L. Truong leg.; **IEBR**. 1♀; HNL2018-036; Vietnam, Dak Lak Province, Chu Yang Sin National Park; 09.v.2018; X. L. Truong leg.; **IEBR**. 2♀; HNL2018-074; HNL2018-075; Vietnam, Gia Lai Province, Kon Chu Rang Nature Reserve; 08.v.2018; X. L. Truong leg.; **VNMN**.

**Non-type material.** 2♀; HNL2018-072; HNL2018-076; Vietnam, Gia Lai Province, Kon Chu Rang Nature Reserve; 08.v.2018; X. L. Truong leg.; NSMT.

***Biasticusgriseocapillus* Ha, Truong & Ishikawa, sp. nov.**

https://zoobank.org/BDAB0B60-64D5-4529-8715-2E1B010AD57B

Figs 5B, 6E–I, 16, 17, 18

**Type material. *Holotype*.** 1♀; HNL2018-038; Vietnam, Dak Lak Province, Chu Yang Sin National Park; 09.v.2018; X. L. Truong leg.; **IEBR. *Paratypes*.** 1♀; HNL2018-037; Vietnam, Dak Lak Province, Chu Yang Sin National Park; 09.v.2018; X. L. Truong leg.; **IEBR**. 1♂; TXL2016-546; Vietnam, Gia Lai Province, Kon Chu Rang Nature Reserve; 28.iv.2016; X. L. Truong leg.; **IEBR**.

**Non-type material.** 1♀; HNL2018-007; Vietnam, Gia Lai Province, Kon Chu Rang Nature Reserve; 05.v.2018; X. L. Truong leg.; NSMT.

***Biasticusluteicollis* Ha, Truong & Ishikawa, sp. nov.**

https://zoobank.org/752E02B7-586A-4A9C-8D70-09DE6199F2EE

Figs 5C, 6J–N, 19, 20, 21

**Type material. *Holotype*.** ♂; HNL2018-025; Vietnam, Dak Lak Province, Chu Yang Sin National Park; 09.v.2018; X. L. Truong leg.; **IEBR. *Paratypes*.** 2♀; HNL2018-020; HNL2018-024; Vietnam, Dak Lak Province, Chu Yang Sin National Park; 09.v.2018; X. L. Truong leg.; **IEBR**. 2♂; HNL2018-085; HNL2018-086; Vietnam, Gia Lai Province, Kon Chu Rang Nature Reserve; 08.v.2018; X. L. Truong leg.; **IEBR**. 1♀; HNL2018-017; Vietnam, Dak Lak Province, Chu Yang Sin National Park; 09.v.2018; X. L. Truong leg.; **VNMN**. 1♀; HNL2018-082; Vietnam, Gia Lai Province, Kon Chu Rang Nature Reserve; 08.v.2018; X. L. Truong leg.; **VNMN**. 1♂; HNL2018-083; Vietnam, Gia Lai Province, Kon Chu Rang Nature Reserve; 08.v.2018; X. L. Truong leg.; **VNMN**.

**Non-type material.** 1♀; TXL2016-616; Vietnam, Dak Lak Province, Chu Yang Sin National Park; 05.v.2016; X. L. Truong leg.; NSMT. 1♂; TXL2016-617; Vietnam, Dak Lak Province, Chu Yang Sin National Park; 05.v.2016; X. L. Truong leg. NSMT; 2♀; HNL2018-018; HNL2018-019; Vietnam, Dak Lak Province, Chu Yang Sin National Park; 09.v.2018; X. L. Truong leg.; IEBR. 2♀; HNL2018-021; HNL2018-023; Vietnam, Dak Lak Province, Chu Yang Sin National Park; 09.v.2018; X. L. Truong leg.; NSMT. 1♂; HNL2018-022; Vietnam, Dak Lak Province, Chu Yang Sin National Park; 09.v.2018; X. L. Truong leg.; NSMT. 1♀; HNL2018-078; Vietnam, Gia Lai Province, Kon Chu Rang Nature Reserve; 08.v.2018; X. L. Truong leg.; NSMT. 2♂; HNL2018-079; HNL2018-084; Vietnam, Gia Lai Province, Kon Chu Rang Nature Reserve; 08.v.2018; X. L. Truong leg.; NSMT. 2♀; HNL2018-080; HNL2018-081; Vietnam, Gia Lai Province, Kon Chu Rang Nature Reserve; 08.v.2018; X. L. Truong leg.; IEBR.
